# Discovering optimal features for neuron-type identification from extracellular recordings

**DOI:** 10.3389/fninf.2024.1303993

**Published:** 2024-02-02

**Authors:** Vergil R. Haynes, Yi Zhou, Sharon M. Crook

**Affiliations:** ^1^Laboratory for Auditory Computation and Neurophysiology, College of Health Solutions, Arizona State University, Tempe, AZ, United States; ^2^Laboratory for Informatics and Computation in Open Neuroscience, School of Mathematical and Statistical Sciences, Arizona State University, Tempe, AZ, United States

**Keywords:** extracellular action potentials, machine learning, neuron-type prediction, simulated EAP, neuron models

## Abstract

Advancements in multichannel recordings of single-unit activity (SUA) *in vivo* present an opportunity to discover novel features of spatially-varying extracellularly-recorded action potentials (EAPs) that are useful for identifying neuron-types. Traditional approaches to classifying neuron-types often rely on computing EAP waveform features based on conventions of single-channel recordings and thus inherit their limitations. However, spatiotemporal EAP waveforms are the product of signals from underlying current sources being mixed within the extracellular space. We introduce a machine learning approach to demix the underlying sources of spatiotemporal EAP waveforms. Using biophysically realistic computational models, we simulate EAP waveforms and characterize them by the relative prevalence of these sources, which we use as features for identifying the neuron-types corresponding to recorded single units. These EAP sources have distinct spatial and multi-resolution temporal patterns that are robust to various sampling biases. EAP sources also are shared across many neuron-types, are predictive of gross morphological features, and expose underlying morphological domains. We then organize known neuron-types into a hierarchy of latent morpho-electrophysiological types based on differences in the source prevalences, which provides a multi-level classification scheme. We validate the robustness, accuracy, and interpretations of our demixing approach by analyzing simulated EAPs from morphologically detailed models with classification and clustering methods. This simulation-based approach provides a machine learning strategy for neuron-type identification.

## 1 Introduction

Classifications based on morphology and spiking dynamics have demonstrated that cortical neurons exhibit several distinct patterns that are robust across cortical areas (Markram et al., 2004, 2015; Spruston, 2008; Gouwens et al., 2019; Kanari et al., 2019). However, the study of waveform features and dynamics of extracellularly-recorded action potentials (EAPs) has lagged. Researchers using EAP features such as trough-to-peak duration or trough half-width have shown that binary classifications (regular/fast spiking or broad/narrow spiking) provide a practical approach to studying putative excitatory and inhibitory single-unit activity (SUA) in cortex (McCormick et al., 1985; Andermann et al., 2004; Barthó et al., 2004; Mitchell et al., 2007; Marques-Smith et al., 2018; Jia et al., 2019). Novel approaches that train machine learning models on additional features (e.g., repolarization slope) have resulted in moderate improvements beyond previous binary classifications (Jia et al., 2019; Trainito et al., 2019). Despite these apparent limitations in identifying neuron-types using extracellular recordings, multiple studies suggest that particular EAP attributes may predictably vary based on the recording location relative to the specific morphology of the neuron (Gold et al., 2006, 2007; Marques-Smith et al., 2018; Teleńczuk et al., 2018; Bakkum et al., 2019).

A convention adopted for single-channel recordings is to analyze EAPs that exhibit a canonical waveform shape (McCormick et al., 1985; Andermann et al., 2004; Barthó et al., 2004; Mitchell et al., 2007; Marques-Smith et al., 2018; Jia et al., 2019). This “canonical” waveform consists of a prominent negative trough correlated with the negative of the first derivative of the intracellular action potential (IAP) at the soma (Henze et al., 2000; Anastassiou et al., 2015; Neto et al., 2016). The negative trough is typically followed by a prominent positive peak associated with the repolarization phase of the IAP. However, this canonical waveform has a non-linear relationship with the recording location along the somato-dendritic axis of the neuron, where displacement from the soma leads to increased contributions from non-somatic morphological domains (Gold et al., 2007; Pettersen and Einevoll, 2008). The increased contribution from different morphological domains influences the ratio of the negative trough to the subsequent positive peak, as well as the time between them (Gold et al., 2007). This is determined by the change in inward and outward currents at specific locations during the different electrophysiological phases of the action potential (Gold et al., 2007). In other words, a negative trough can be accounted for by the inward sodium or calcium current phase and a positive peak accounted for by an outward potassium current phase. This phase-based analysis can be extended even further. The appearance of a prominent positive peak prior to a negative trough indicates a large contribution of a local capacitive current to the EAP waveform (Gold et al., 2007; Teleńczuk et al., 2018). Moreover, the variability in amplitude of simulated EAPs at fixed distances relative to the soma is explained by variability in total cross-sectional area of neurites where they meet the soma (Pettersen and Einevoll, 2008). It is plausible that such relationships could be exploited to detect asymmetries in neurite geometry for single neurons.

Because spatiotemporal profiles of EAPs vary with electrode location and cell type, it is not possible to infer neuron-type from the snapshot obtained in a single-channel recording. However, given that subcellular differences impact phase-based features of EAP waveforms, the use of high-density recording probes has an important advantage over single-channel probes. Multichannel recordings maintain the spatial relationships among isolated EAPs, and these spatial relationships, in principle, hold additional information about neuron-types. In an experimental study using high-density recordings, Jia et al. (2019) detect backpropagation across multiple channels and demonstrate that spatiotemporal features, such as waveform amplitude spread and the velocity of the EAP trough above and below the soma, are important features for identifying putative neuron-types. Moreover, both experiments and neuron modeling studies show that spatial patterns of EAPs reflect information about the spatial relationship between neuron and recording probe (Somogyvári et al., 2005, 2012; Szymanska et al., 2013; Delgado Ruz and Schultz, 2014; Buccino et al., 2018; Marques-Smith et al., 2018), the orientation of the somato-dendritic axis (Somogyvári et al., 2012; Delgado Ruz and Schultz, 2014; Buccino et al., 2018), gross morphology (Somogyvári et al., 2012; Delgado Ruz and Schultz, 2014; Buccino et al., 2018), and nearby morphological domains (Gold et al., 2007; Radivojevic et al., 2016; Teleńczuk et al., 2018). Thus, subcellular differences in neuronal electrophysiology and morphology are observable across EAPs centered around the soma.

These results illustrate a gap in studies of neuron classification based on morphology and fast timescale electrophysiology. We address this gap by systematically studying sources of spatiotemporal EAP waveforms (*EAP sources*) from simulated extracellular recordings. Here, detailed biophysically-based neuron models are simulated using computational protocols that mimic high-density, linear probe configurations. These computational models provide repeatability, the ability to look at many different neuron-types, and the possibility to link EAPs to details of the neuron morphology and membrane mechanisms. Distinct spatial and multi-resolution temporal patterns are discovered using a combination of computational modeling, automated feature engineering, and machine learning.

**Figure 1:**
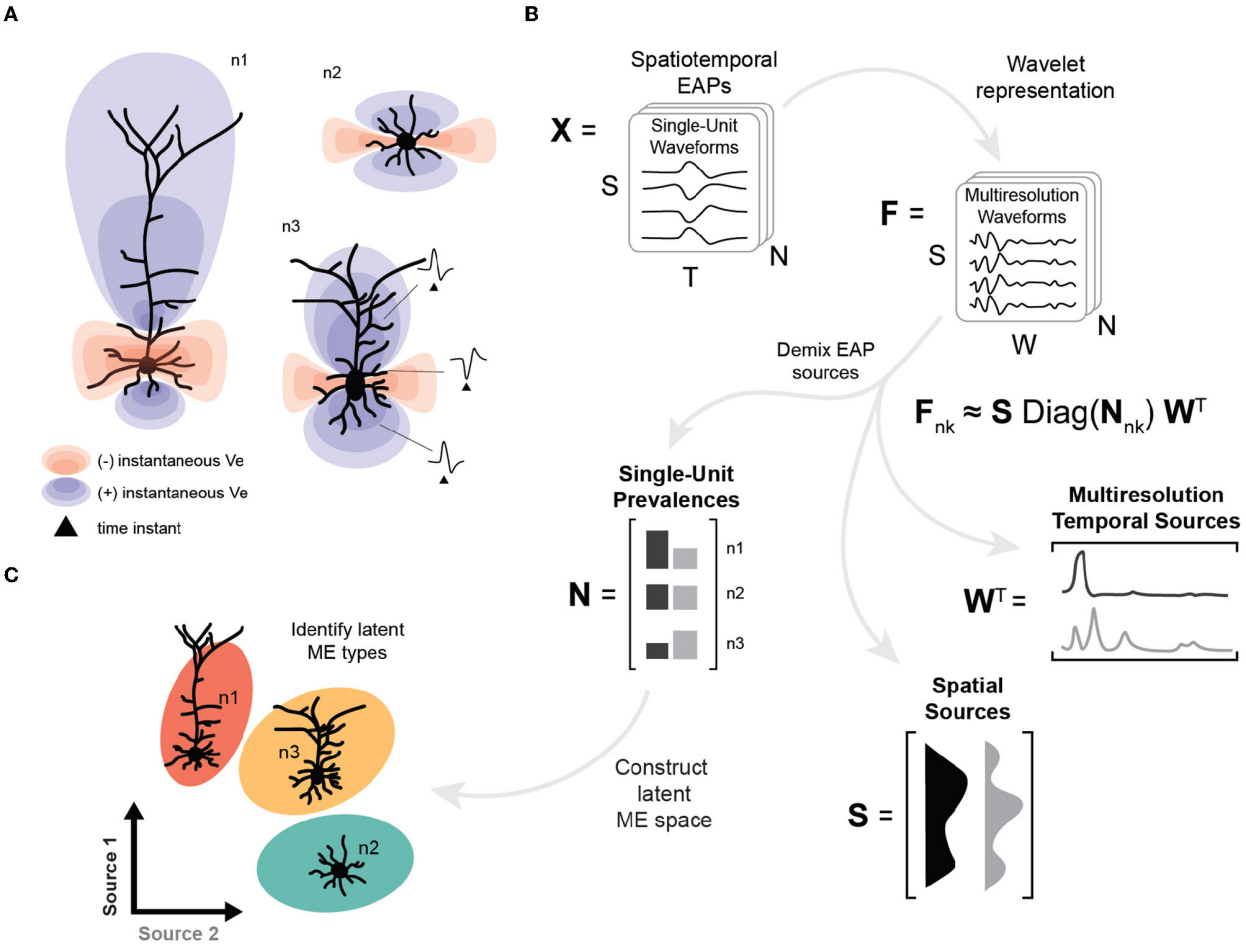
High-level overview of neuron-type identification strategy. **(A)** Cartoon of instantaneous extracellular potential of canonical waveforms at time of spike. The shape, extent, and polarity at different locations of EAPs reflect differences in morphology. **(B)** Schematic of demixing using spatiotemporal EAP waveforms, **X**, from multichannel recordings with S channel locations, T time points, and N recorded units associated each of the probe locations for each model. The inputs to the demixing step are the W coefficients for the multiresolution wavelet representation for each channel and unit, denoted by **F**. Demixing reveals multiple EAP sources describing how factors (space, time, and units) contribute to EAP waveforms. Each EAP source describes the contribution of a pattern across spatial locations, **S**, as well as the corresponding timescales and times, **W**. The remaining factor **N** describes the prevalence of the corresponding sources across all units. The prevalences of these sources for each unit (*n*1, *n*2, *n*3) provide input features for neuron-type identification. **(C)** Construction of the latent morpho-electrophysiological (ME) space relies on **N** where differences in the prevalence of sources are correlated with local morphometrics (like cross-sectional area) and gross morphological properties (such as location of terminal ends of basal dendrites).

EAP sources are typically mixed due to the superposition of EAP waveforms from subcellular domains of neuron morphologies (Figure 1A). In this study, we apply tensor components analysis (Williams et al., 2018) to features extracted through multiresolution wavelet analysis (Mallat, 1989; Quiroga et al., 2004) to demix the relative prevalence of these EAP sources in simulated extracellular recordings (Figure 1B). The result is a low-dimensional representation for recorded units that describes how EAP sources vary across single-units, relative recording site, and phases of the action potential (Figure 1B, bottom). Here, we find that four EAP sources best characterize waveform patterns shared across a diverse population of cortical neuron-type families. We demonstrate how the demixed representations can be used as features to predict population-level distributions of morphological properties and to identify reliable morpho-electrophysiological (ME) types given biophysical constraints for recordings of SUA (Figure 1C). Finally, we show how our demixing strategy performs on the problem of unsupervised discovery of excitatory and inhibitory neuron models.

Our systematic, data-driven pipeline uses an unsupervised method to extract patterns spanning channels and timescales while also representing individual units. We simulate extracellular recordings of diverse cortical neuron-type models at many spatial locations. We find multiple unique spatial templates where combinations of these templates provide representations of single-units that are predictive of variations in the underlying neuron-types, and we link these to morphology. These results can provide higher precision analyses for *in vivo* extracellular electrophysiology recordings given the fundamental constraints of the field's existing techniques.

## 2 Methods

All analyses and simulations are implemented in the Python programming language. Standard Python modules that are critical for the pipeline include: Numpy (Harris et al., 2020), Pandas (McKinney, 2020), Scipy (Virtanen et al., 2020), and Scikit-Learn (Pedregosa et al., 2011).

### 2.1 Simulations of neuron-type models

Neuron-type models were obtained from the NeuroML Database (NeuroML-DB.org) using the database API (Birgiolas et al., 2023). The database hosts single neuron models in multiple model formats and provides numerical details for running models. Models were downloaded in the HOC format for simulation and the NeuroML format for morphometric analyses. We chose models that were previously developed from studies of rat primary somatosensory cortical neurons as part of the Blue Brain Project (Markram et al., 2015). NeuroML-DB model IDs for these models are provided in Supplementary Table 1. Each multicompartmental model consists of multiple, detailed morphological domains: soma, axon initial segment (AIS), basal dendrites, and possibly, apical dendrites. The AIS for each model is composed of two compartments of equal length and diameter. One compartment is the stem of the axon and is connected to the soma. The other is connected to the stem compartment and extends in the same direction as the stem. The dendritic domains are highly detailed, consisting of hundreds or even thousands of compartments. In total, we obtained 105 neuron models—five morphological variants for 21 neuron-type families. These families include 11 types of excitatory pyramidal cells and 10 types of inhibitory interneurons. These model families were chosen due to their availability, detail, and development within one research laboratory.

The neuron-type models were simulated using the Python package NetPyNE (Dura-Bernal et al., 2019) with the NEURON simulator (Carnevale and Hines, 2006). The model-based bias current amplitude and rheobase were obtained from the NeuroML Database (Birgiolas et al., 2023). Steady state membrane potentials were attained by simulating a bias current injection for 1,000 *ms* prior to applying a 50 *ms* duration current at 3x the rheobase to initiate an action potential.

**Figure 2:**
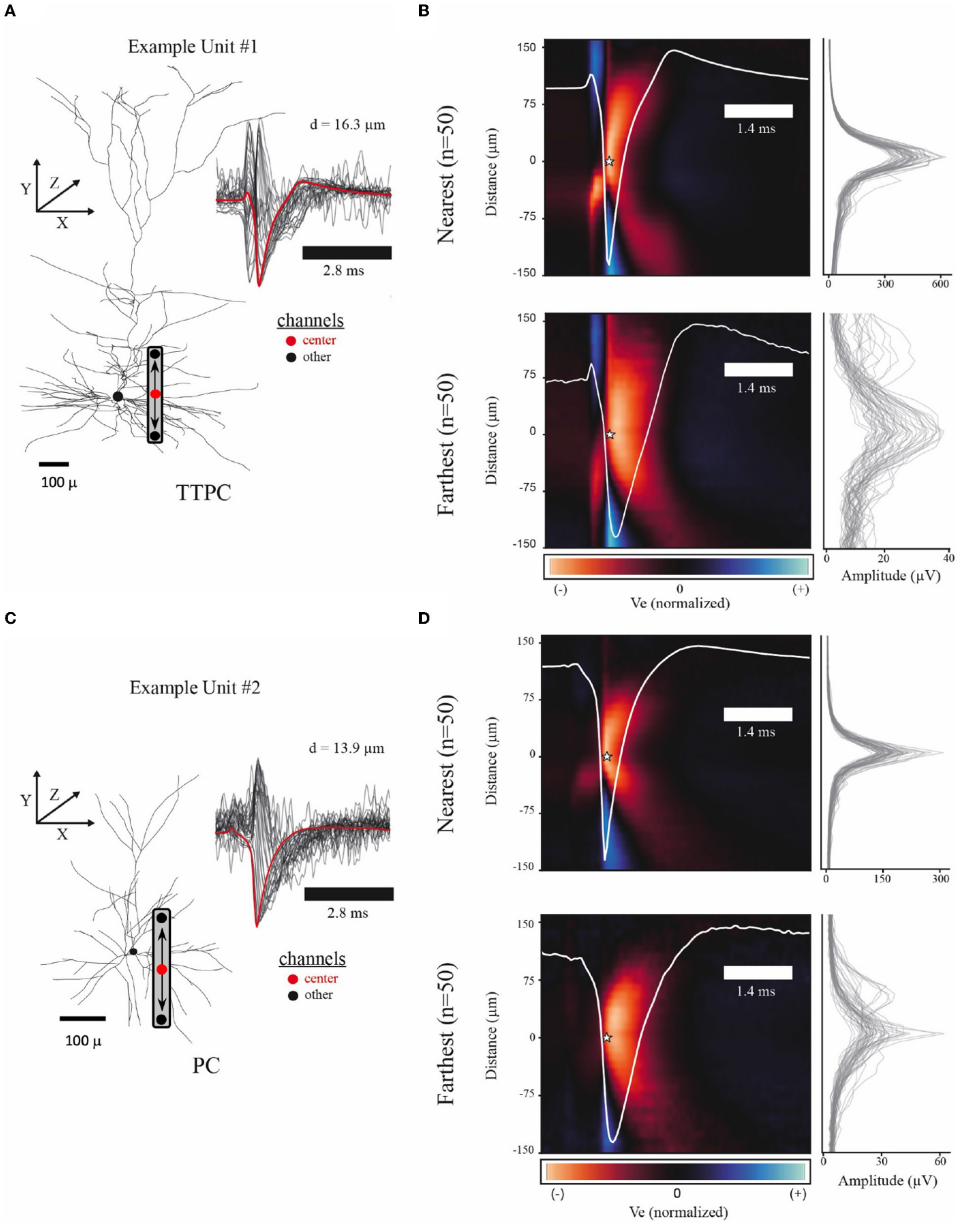
Example spatiotemporal EAPs from simulated single-units. **(A)** Example model morphology from the thick-tufted pyramidal cell (TTPC) family. Inset shows EAP waveforms from 31 adjacent channels (black) with center channel (red) at a radial distance of 16.3 microns from the soma. Schematic of probe placement shows the location of the center channel (red dot) and the orientation of the channels above and below center. **(B)** Colormap shows average EAP waveforms from the 50 closest (top, *d* = 10.41–16.28 microns) and farthest (bottom, *d* = 65.74–154.24 microns) units across the TTPC neuron-type family. EAPs were normalized before averaging. Color scale adjusted to the maximum fluctuation of the EAP. Star indicates the center channel and the intracellular spike time for all units. Center waveform depicted in white. Amplitudes for each channel used to normalize EAPs (right) show amplitude spread for the *n* = 50 units. **(C)** Similar to **(A)** showing superficial pyramidal cell (PC) family. **(D)** Similar to **(B)** showing closest (top, *d* = 10.23–13.88 microns) and farthest (bottom, *d* = 41.10–74.67 microns) units across PC neuron-type family.

For each model, the extracellular space was defined as a 3D grid centered at the soma. The *Y*-axis was oriented with positive and negative values reflecting more superficial and deeper locations relative to the soma, respectively (Figures 2A, C). In other words, the *Y*-axis reflects relative position of the soma along the cortical depth. Similarly, the XZ-plane was defined perpendicular to the *Y*-axis and centered at the soma. Extracellular potentials were computed within the 3D space using built-in functions from NetPyNE. NetPyNE uses a transmembrane current-based forward modeling scheme (Agudelo-Toro, 2012; Lindén et al., 2014).

### 2.2 Constraining extracellular space

In typical extracellular recording studies, single units are often excluded if they do not meet an amplitude threshold sufficient to overcome background noise (Gray et al., 1995; Henze et al., 2000; Segev et al., 2004). In this study we first wanted to identify the region of modeled extracellular space where simulated EAPs are consistent with experimental conditions. To that end, we characterized the detectability of neuron-types and identified those with EAP amplitudes <20 μ*V* at distances of ~10 μ*m* from the soma. This detectability threshold was conservative relative to the accepted convention of using signal-to-noise levels that are above a value that is 3–4 times the noise floor or 50–60 μ*V* (Abeles, 1991; Henze et al., 2000; Segev et al., 2004). Thus, simulation studies of EAPs were performed in two stages: (1) EAPs were simulated to uncover the detectability limit for each neuron-type model and (2) EAPs for the studies described here were simulated within this detectability limit.

#### 2.2.1 Detectability limit

For the first stage, using a forward modeling scheme for each of the models, we computed the extracellular potential, *V*_*e*_, at 1,050 locations. The XZ-coordinates of these locations were circularly distributed at 10 angles along the XZ-plane that were chosen uniformly at random. For each angle, we used 21 radial distances from the origin along the XZ-plane, ranging between 10–120 μ*m* at 5 μ*m* increments. This process was repeated, resulting in radial grids at 20, 10, 0, −10, and −20 μ*m* from the origin along the *Y*-axis. Each neuron-type model was subjected to the current injection protocol described above. We then computed a detectability limit for each separate model based on amplitudes computed from individual EAPs at these 1,050 locations. Here, the amplitude was taken to be the absolute difference between the trough and peak of an EAP, where the trough and peak were defined as the minimum and maximum values of an EAP, respectively.

The detectability limit was taken to be the average radial distance resulting in an amplitude of at least 20 μ*V*. The average detectability range across all neuron-type models was ~35 μ*m* (μ = 35.48, σ = 14.71). By fitting the average maximum amplitudes to exponential curves, <2% (2/105) of models were unable to generate EAPs with amplitudes >20 μ*V* at locations one micron from the soma. These two models were from the Layer 2/3 small basket cell and Layer 2/3 double bouquet cell neuron-type families and had peak amplitudes of 8.4 and 1.95 μ*V*, respectively. We excluded these two models from subsequent analyses.

#### 2.2.2 Simulating EAPs for further study

The model-specific detectability limits for the remaining 103 models from 21 neuron-type families were used to construct the simulation dataset used for all further analyses. For each model, we first chose 100 different locations where we place the linear probe for recording simulated EAPs. For these locations, the radial distances from the origin in the XZ-plane were uniformly distributed between 10 microns and the associated detectability limit. The angle for each location was also selected from a uniform distribution. Each of these 100 locations were taken as the center of a 64-channel linear probe with channel locations spaced every 10 μ*m* along the *Y*-axis, where center channels were jittered using a uniform distribution with bounds of ±5 μ*m* along the *Y*-axis centered at the origin. The current injection protocols described above were used to generate EAPs at channel locations. Additionally, we recorded the somatic membrane potential for each simulation.

### 2.3 Waveform pre-processing

Action potentials are classically divided into intervals, or phases, of interest. These intervals are associated with the depolarization, repolarization, and recovery phases, reflecting the activation and inactivation of inward and outward currents. A non-classical phase of interest is the capacitive phase associated with spike initiation in the axon initial segment and propagation into the soma (Gold et al., 2006; Radivojevic et al., 2016; Teleńczuk et al., 2018). The effects of these phases also can be seen in EAPs (Figure 5A). Using the average EAP (white curve, bottom panel Figure 5A), we determined the average spike width (~1.4 *ms*), defined as the time between the trough and following peak. The duration of snippet windows for further analysis of EAPs was taken to be a multiple of the average spike width. To include all phases of interest, the extracellular action potential was extracted using a total snippet window of 5.6 *ms* with a 1.4 *ms* pre-spike interval, where the spike time is defined as the time the somatic membrane potential achieved its peak value during an action potential. The extracted spike snippets were then used to construct the mean spike-aligned waveform.

Extracellularly recorded signals around a neuron appear to exponentially decay with recording distance (Rall, 1962; Gray et al., 1995; Segev et al., 2004; Pettersen and Einevoll, 2008). Thus, recorded extracellular action potentials have signal-to-noise ratios that decrease as background activity and electrical noise become more prominent with increasing distance. This is the case for both probes situated at large distances from the soma, as well as, more distally located channels within probes situated closer to the soma. In order to capture these noise relationships, we modeled the noise component of extracellular action potentials as Gaussian distributed noise with a 1/*f* power law spectrum, where *f* is the frequency. A total of 200 independent noise signals were generated for each channel, then scaled and averaged to match noise levels from Neuropixel recordings of rat somatosensory cortex (Marques-Smith et al., 2018). This process was independently repeated across all channels resulting in uncorrelated background noise. Finally, each channel's waveform was shifted by subtracting the median of the waveform.

### 2.4 Selecting canonical multichannel waveforms

Typically, studies using multichannel recordings of EAPs with linear probes designate a single channel as nearest to the soma then orient additional channels symmetrically relative to this channel, i.e., centering the waveform along the probe. Waveforms are often excluded from analysis if they do not exhibit a canonical shape, i.e., if the waveform does not exhibit a prominent negative trough preceding a prominent positive peak. Our goal is to select centered, canonical waveforms for further analysis.

Usually, the channel designated as the center channel is the channel with the largest amplitude waveform (Buzsáki and Kandel, 1998; Somogyvári et al., 2005, 2012; Delgado Ruz and Schultz, 2014; Jia et al., 2019). However, high-resolution empirical findings and modeling of EAPs near the axon initial segment (AIS) indicate that the current dipole created by AP propagation from the AIS to the soma contributes more to the amplitude of the EAP than the soma alone (Teleńczuk et al., 2018; Bakkum et al., 2019). Thus, the largest amplitude waveforms occur between the distal end of the AIS and the soma.

Because we know the location of the channel closest to the soma in these modeling studies, we also assessed the reliability of selecting the channel with the largest amplitude waveform as the center. This process is complicated by the fact that there may be multiple channels with comparable large amplitudes. For example, this can occur when the center channel is sufficiently far from the soma but a different channel is close to the axon initial segment (AIS). In this case, the waveforms from the channel located close to the axon initial segment have spike troughs that occur earlier than the intracellular voltage peak used for spike detection. With this in mind, we developed a set of heuristics to center multichannel waveforms using the amplitude profile from across a linear probe (Figures 2B, D). We collected each peak in the amplitude profile that exceeded 60% of the maximum amplitude of the probe. If there was only a single peak, we took this to be the center channel. If there were high amplitudes associated with waveforms from multiple channels, we considered the two locations with the largest peak. The two waveforms were characterized as inverted or not, based on *V*_*e*_ at the intracellular spike time. If the potential at the spike time was greater than the median value of the waveform, then the waveform was taken to be inverted and rejected as the center channel. After the center channel was selected, we retained 31 channels for further analysis—the center channel along with 15 channels above and 15 channels below.

Spike detection often involves defining a negative threshold for recorded extracellular potentials and taking the nearby trough as the spike time. Since we have access to the intracellular spike time, the selected center channel EAP waveforms were classified as canonical or non-canonical based on potential values around the intracellular spike time (*t* = 0 ms) so that non-canonical waveforms could be excluded from further analysis. In a typical experimental setting, such waveforms would not be excluded from analyses if detected; however, we made this choice to ensure that the center channel waveform was not dominated by the axonal spike nor inverted due to the soma-AIS current dipole (Teleńczuk et al., 2018). The known spike time was compared to the time when the extracellular potential achieved its minimum value. We computed upper and lower tolerances based on the mean and standard deviation of the waveform (μ±σ). We defined waveforms as non-canonical if they: (1) had a prominent positive deflection greater than the upper tolerance during the pre-spike interval (−0.21 to 0 ms), (2) had a minimum value that occurred earlier than the pre-spike interval, or (3) did not have a prominent negative deflection less than the lower tolerance during the post-spike interval (0–0.42 ms). Non-canonical waveforms comprised <3% of all waveforms based on these heuristic criteria.

Finally, to ensure the same number of simulated probe locations for each neuron-type family in our further analysis, the final dataset consisted of 400 probe locations for each neuron-type family. These locations were chosen randomly from all simulated data associated with a given neuron-type family with center channel waveforms that have the canonical waveform shape.

### 2.5 Demixing steps

Next we use a machine learning approach to demix the underlying sources of these spatiotemporal EAP waveforms. Our demixing approach relies on a multiresolution wavelet representation of the waveforms followed by tensor decomposition. Here we describe these steps in more detail.

#### 2.5.1 Organization of the data

Simulated EAPs were organized into a single tensor with three axes (Figure 1B). The first axis corresponds to the relative spatial locations and has dimension *S* = 31 for the number of channels associated with the simulated probe. The second axis corresponds to the times associated with each EAP and has dimension *T* = 128 for the number of time points for each recorded waveform. As described above, we generate data of dimension *S*×*T* for each probe location for each model, which we now refer to as a *single-unit* or simply *unit*. The third axis corresponds to the final set of units across the multiple neuron-type models and has dimensions *N* for the total number of units (*N* = 400 × 21). We use **X** to denote the data tensor with dimensions *S*×*T*×*N*.

#### 2.5.2 Wavelet representation

Most feature analyses of single-channel EAP waveforms rely on readily apparent waveform features, such as trough-to-peak duration or slope during an interval of interest, but this approach limits the analysis to the coarsest of timescales. In order to discover patterns that are observable across multiple spatial locations but possibly hidden within different timescales, we use a multiresolution wavelet representation for each waveform. Such representations have been shown to result in optimal spike sorting when used as features for clustering (Quiroga et al., 2004). Automated feature extraction is applied to each of the *S* rows of **X** producing a wavelet-based feature representation of dimension *W* for each single-channel waveform. This feature representation is denoted by the tensor **F** with dimensions *S*×*W*×*N*.

**Figure 3:**
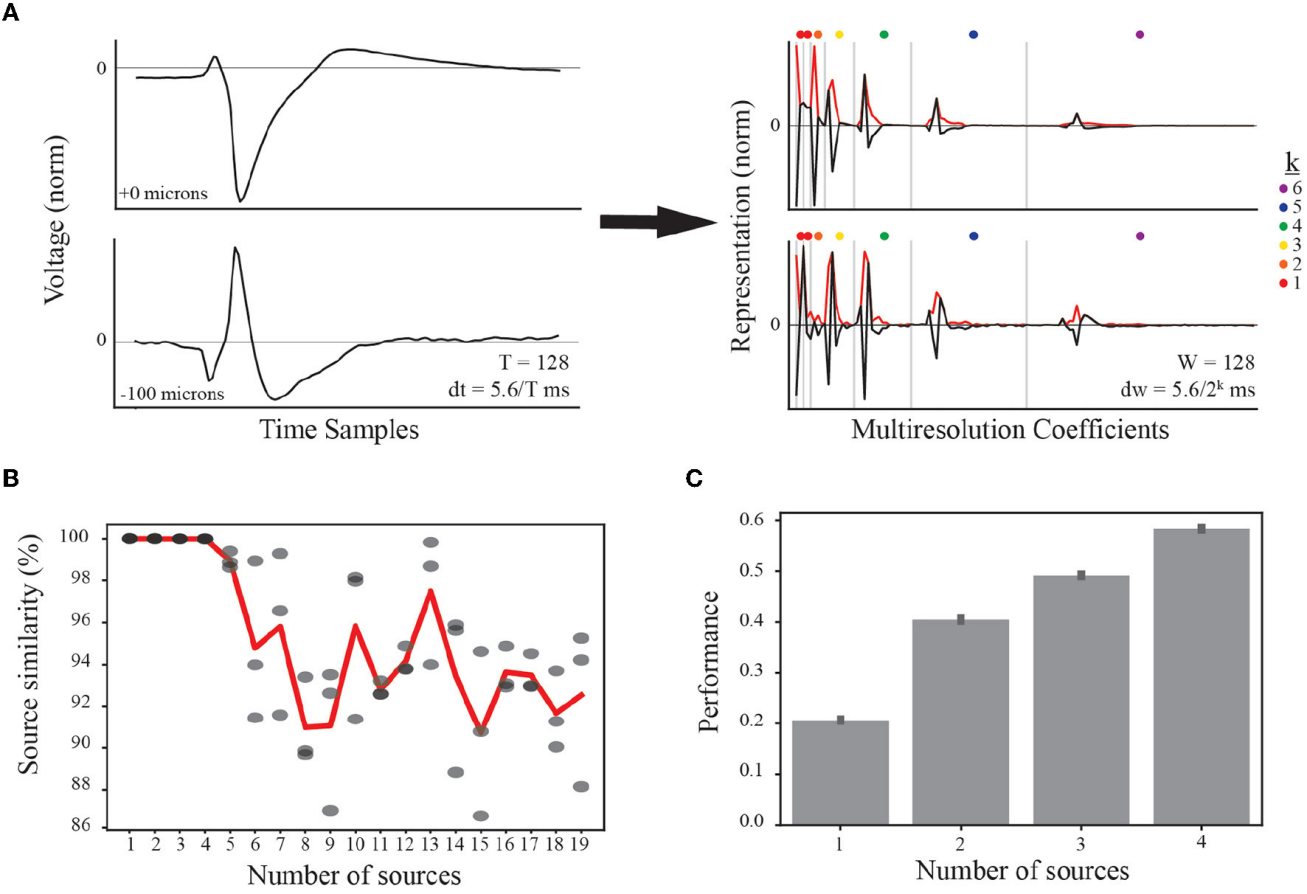
Feature representation and selection of number of EAP sources. **(A)** (left) Normalized EAP waveforms at two example channel locations from the same simulated unit illustrate a canonical waveform (top) and an associated inverted waveform (bottom). Such waveforms indicate a common source, e.g., a soma-axon current dipole. As indicated in the text, each temporal waveform represents 5.6 ms. (right) Multiresolution analysis using Haar wavelets results in multiresolution wavelet representations (black curves) shown grouped by resolution levels (vertical gray bars). Resolution at each level between gray bars is given by dw where the exponent k is indicated by the dot color legend. Each level consists of multiple coefficients ordered in time and corresponding to the convolution of the Haar wavelet associated with that level. The absolute values of the multiresolution wavelet representations are used as the input to TCA (red curves). **(B)** When performing TCA, we must select the parameter value for the number of sources, *R*, based on similarity and performance. The result of TCA is a set of matrices with dimensions *S*×*R*, *W*×*R*, and *N*×*R*. For each source count parameter value from 1 to 19, four independent demixings are trained to the data. The model with the lowest reconstruction error is taken as the target model for computing similarity scores among the remaining three models (similarity scores gray circles). Source counts are selected for further testing if the average source similarity (red curve) is >99%. **(C)** Each source count with sufficient similarity is used to classify morphological classes using a random forest classifier, and the performance accuracy for each is plotted with 95% bootstrapped confidence intervals.

Our multiresolution wavelet analysis is implemented using the PyWavelets Python package (Mallat, 1989; Lee et al., 2019) and requires a choice of mother wavelet and number of levels. We use the Haar wavelet because it is real-valued with both orthogonal and symmetric properties. This symmetry enables similar representation of EAPs generated by current source dipoles (e.g., axon-soma dipoles, Teleńczuk et al., 2018) that contribute to waveform inversion (Figure 3A, left). Further, this symmetry is conserved by taking the absolute value of all multiresolution coefficients (Figure 3A, right). At each level, the multiresolution wavelet analysis reduces the length of a waveform by half in an iterative process. The total number of levels was determined based on physiological intervals of interest relative to the spike time, including the prespike, depolarization, repolarization, and recovery associated phases of interest. In our final analysis, we include additional levels to capture less apparent, faster timescale subdivisions. **F** is the input for the tensor decomposition at the next stage of analysis.

#### 2.5.3 Tensor decomposition

Next we applied tensor components analysis (TCA) to the multiresolution wavelet representation, **F**. TCA is a higher-dimensional generalization of principal components analysis with special properties to optimally handle tensor data and demix data generated from mixed contributions originating from multiple, non-disjoint sources (Harshman, 1970; Williams et al., 2018). TCA has the additional benefit of not requiring orthogonality, and it allows for interpretations of results in terms of the original axes of the feature representation.

Our goal is to use TCA to demix EAP sources across the population of units. We implemented TCA as a non-negative, canonical polyadic (CP) tensor decomposition with the block coordinate descent (BCD) method using the Python package TensorTools (*tensortools.ncp_bcd*, Williams et al., 2018). This decomposition results in *R* non-orthogonal components (**s**^*r*^, **n**^*r*^, and **w**^*r*^ for *r*∈1, …, *R*) that approximate the feature representation as


$$f_{i,nk,j}\approx \overset{R}{\underset{r=1}{\sum }}s_{i}^{r}n_{nk}^{r}w_{j}^{r},$$

where *i*∈{1, 2, ..., *S*}, *nk*∈{1, 2, ..., *N*}, and *j*∈{1, 2, ..., *W*} (see Figure 1B). Here, the column vectors **s**^*r*^ and **w**^*r*^ for a given *r* represent the population-level spatial distribution of an EAP source across single-units and the relative contribution of the EAP source across multiple timescales, respectively (Figure 1B). The column vector **n**^*r*^ represents how prevalent a given EAP source is for each of the N single-units. Finding the neuron-type source average using the $n_{nk}^{r}$ values for all single-units belonging to the same neuron-type family yields a neuron-type representation in terms of the EAP sources and thus represents the probability that the EAP sources would be observed for that neuron-type. The reconstruction error and decomposition similarity scores were also computed using TensorTools and tested for multiple values of *R* (Supplementary Figure 1).

**Figure 4:**
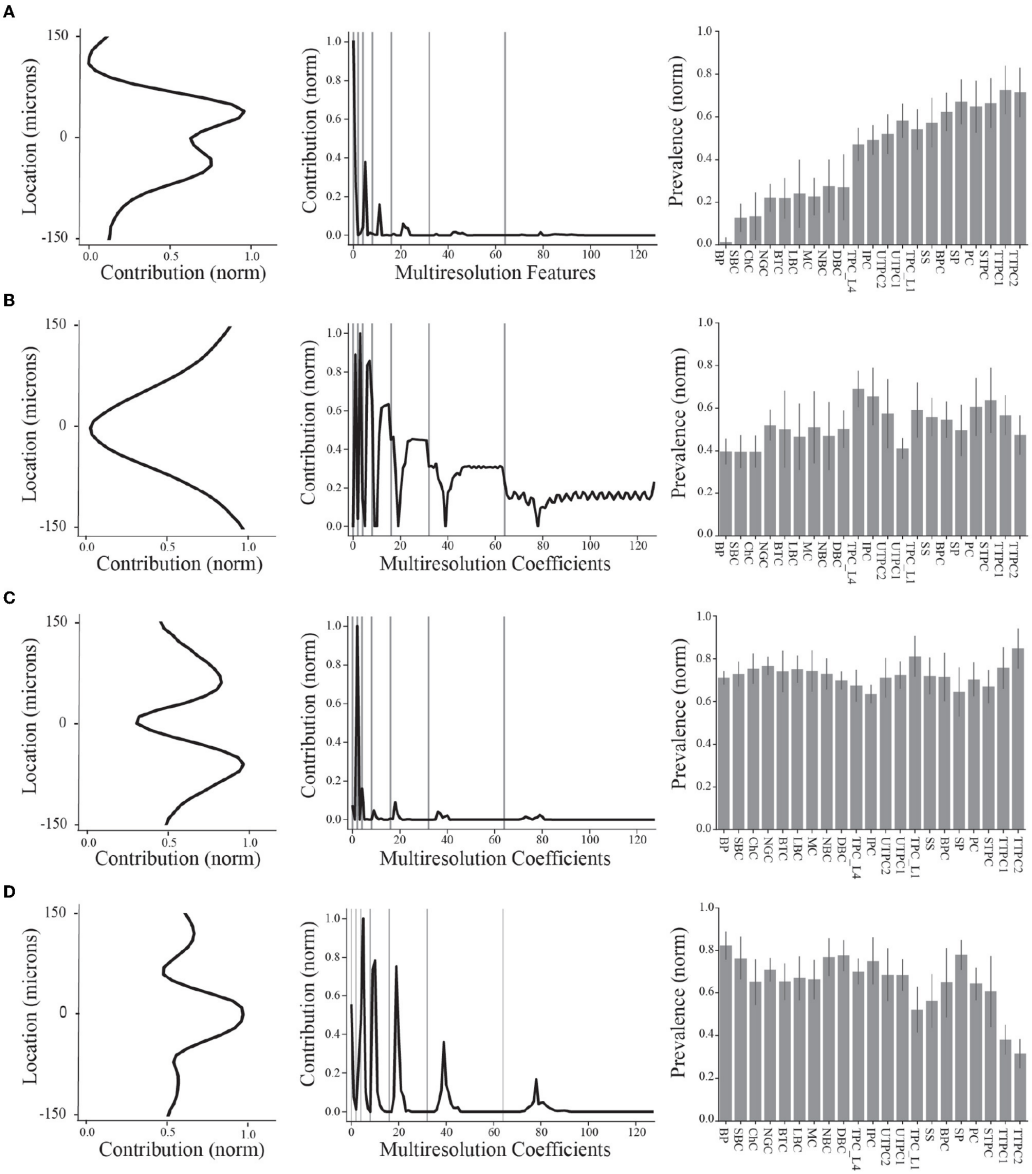
Summary of final demixed EAP sources. **(A–D)** The normalized contribution of each EAP source at the 64 channels along the probe. The center channel is taken to be closest to the putative perisomatic region of all neuron models. The multiresolution temporal sources (middle) describe the contribution of each EAP source across multiple timescales (black curves, gray lines show grouped timescales), as in Figure 3A. In other words, the values of each spatial and multiresolution temporal source reflect the contribution of that EAP source in space and time to the EAP waveforms. Source prevalences (right) were averaged within neuron-type families. Error bar depicts 1 standard deviation. Shown prevalences were normalized by their maximum values.

**Figure 5:**
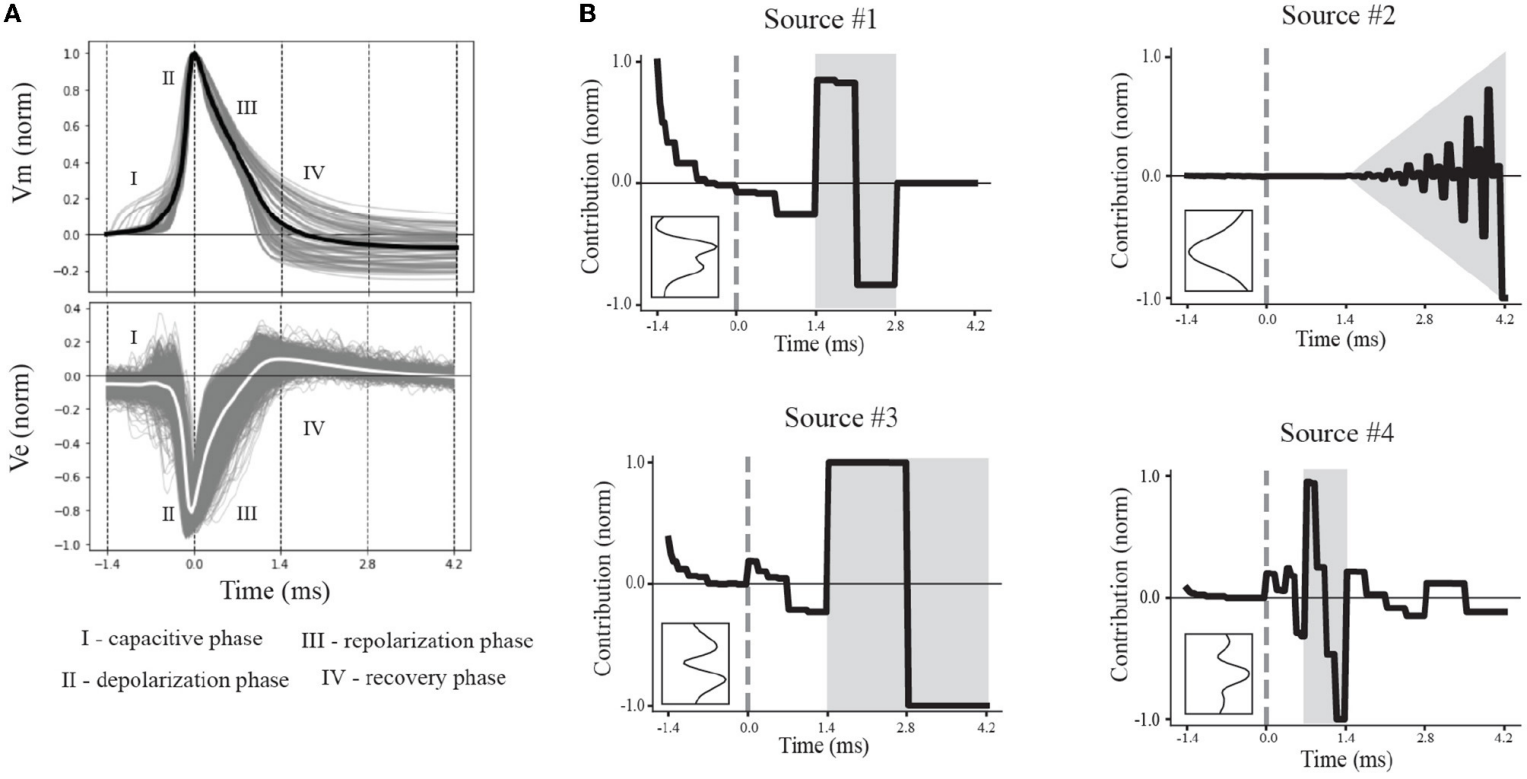
Multiresolution patterns reveal underlying electrophysiological phases. **(A)** Normalized canonical IAP (top) and EAP (bottom) waveforms show variability within electrophysiological phases of interest (I–IV). The average (black) of the action potentials represent the canonical IAP shape. The corresponding EAPs (gray) and canonical EAP (white) at the center channel across the probes (bottom) are labeled based on the phases of the canonical IAP. **(B)** The contributions of the multiresolution temporal sources (right) were reconstructed, yielding their temporal dynamics, to illustrate the contribution relative to the spike time (gray dashed line). Intervals with larger contributions of multiresolution temporal sources are depicted in gray. Insets show the corresponding spatial sources.

In what follows, the column vectors **s**^*r*^ and **w**^*r*^ are termed *spatial sources* and *multiresolution temporal sources*, respectively. When referring to the spatial and multiresolution temporal sources corresponding to the same *r*, we use the term *EAP source*. The numerical values of these two components are referred to as their *contributions*, e.g., the spatial source **s**^1^ has a contribution of ${\text{s}}_{i}^{1}$ at the *i*^*th*^ channel. Thus, in our results, we show contributions for both spatial courses (Figure 4, left) and multiresolution temporal sources (Figure 5B). In contrast, the column vectors **n**^*r*^ are termed the *single-unit source prevalences* and their numerical values are referred to as the *prevalence* for the corresponding unit.

### 2.6 Morphometric analysis

All morphometrics were computed from the NeuroML format description for each model (Gleeson et al., 2010), where each reproduces the morphological description from the original Blue Brain Project model (Markram et al., 2015). This XML-based language enables flexible, automated extraction of compartment details using the ElementTree XML API in Python, as well as computation of model morphological features. NeuroML specifies the structure of morphologies within a nested hierarchy of parent-child relationships between compartments (Crook et al., 2007). Each compartment (segment element) is specified by the *xyz*-coordinates and radius for the proximal and distal ends of the compartment. All neuron-type model segments were specified as belong to somatic, axonal, basal dendrites, or apical dendrites segment groups, using the segment labels provided in the original Blue Brain Project models.

**Figure 6:**
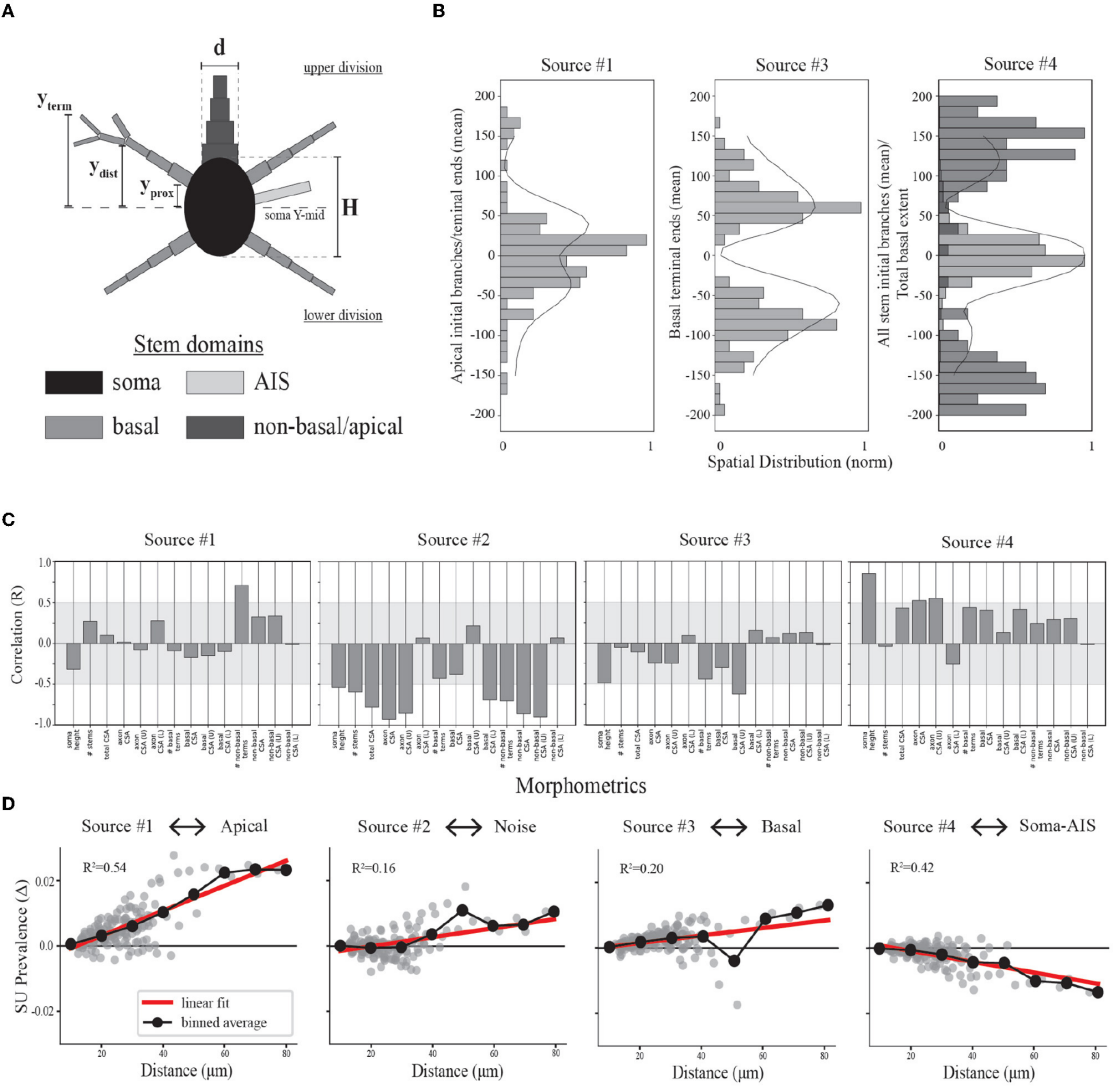
Relationships between model morphologies and demixed EAP sources. **(A)** Schematic of multi-compartment models showing different morphological domains. **(B)** Bar plots depict spatial profiles for different stem geometries relative to discovered spatial sources (as depicted in Figure 4, left column). The spatial sources are scaled to illustrate shared patterns. **(C)** Bootstrapped canonical correlation analysis shows the relationship between morphometrics and SU source prevalences. (CSA, cross sectional area; U, upper; L, lower). **(D)** Scatter plots illustrate the differences in SU source prevalences across recording distances, radial distance **r** along XZ-plane, for each neuron-type family and relative to the nearest recording distance (~10 microns for each). Linear regression (red) and binned averages (black, bin size = 10 microns) indicate whether the source tended to increase or decrease with distance from the putative perisomatic region.

We computed morphological features of the most proximal compartments of the neuron model for use in further analysis. This approach is based on modeling results demonstrating that overall differences in the cross-sectional area and distance from the soma of local neurites are reflected by the amplitude variability of EAPs (Pettersen and Einevoll, 2008; Teleńczuk et al., 2018). For the somatic group, we computed the overall soma height in the *Y*-dimension (i.e., depth axis of cortex), the number of segments with the soma as a parent (known as stems), and the total cross-sectional area (CSA) of all stems. The CSA was computed as π(*d*/2)^2^ where *d* was the diameter of the proximal end of the stem (Figure 6A). Many neuron-types have multiple basal dendritic stems that project in several directions. We divided the basal stems into upper and lower divisions by bisecting the somatic segments into upper and lower groups based on the soma mid-point in the *Y*-dimension (Figure 6A, *soma Y-mid*). For the basal dendrite group, we computed the total number of basal stems, the total CSA of all stem segments as well as the CSA for the upper and lower stem divisions. We also computed the average *Y*-locations of the basal terminal ends (Figure 6A, *y*_term_) for the upper vs. lower stem groups. Those neuron-type models that have apical domains have at most two, projecting upward, downward or in both directions. We also computed the CSA of the upper and/or lower apical stems. For the axonal group, we computed the stem CSA of the most proximal segment in addition to noting whether it belong to the upper or lower division of soma. Finally, we determine the proximal and distal *Y*-locations of each neurite stem (Figure 6A, defined as compartments starting at the soma and extending up to the first branch, *y*_prox_ and *y*_dist_) as well as the distal *Y*-locations of neurite terminals (Figure 6A, *y*_term_).

### 2.7 Bootstrapped canonical-correlation analysis

Canonical correlation analysis (CCA) is a cross decomposition method for finding the linear combination of two random variables that maximizes correlations between the variables. We applied CCA to determine how predictive the single-unit source prevalences were of local morphometrics. To implement CCA, we use the SciKit-Learn *cross_decomposition* library in Python. We fit *R* independent CCA models to the matrix of morphometrics for each neuron-type model, **M**, and the matrix of randomly selected single-unit source prevalences for each neuron-type model and corresponding to source *r*, **P**^*r*^ for *r*∈1, …, *R*. For all CCA models, we took *n_components* to be 1. We adopted a bootstrapped approach to CCA where the matrices **M** and **P**^*r*^ were independent constructed on each bootstrap iteration. We describe the matrices, their construction and bootstrap algorithm below.

Let **M** be the *X*×*M* matrix of morphometrics for each neuron-type model where the total number of neuron-type families is given by *X* and the total number of morphometrics to be predicted is given by *M*. In other words, $m_{i}^{j}$ of matrix **M** is the *j*^*th*^ morphometric of the *i*^*th*^ neuron-type model. Let **P**^*r*^ be the *X*×*P* matrix of single-unit source prevalences where *P* is a fixed number of source prevalences that are randomly selected from the total number of source prevalences corresponding to the same neuron-type model and *r* corresponds to the *r*^*th*^ source, as above. In this case, $p_{i}^{k}$ of **P**^*r*^ corresponds to the *k*^*th*^ source prevalence of source *r* and the *i*^*th*^ neuron-type model.

At every bootstrap iteration, one neuron-type model is randomly selected from each of the *X* neuron-type families. The matrix of morphometrics **M** is then populated by the *M* morphometrics of the *X* selected neuron-type models. For the source prevalence matrix, we populated the matrix **P**^*r*^ with 50 randomly sampled (with replacement) source prevalences for each of the 21 selected neuron-type models and demixed source *r*. Matrices **M** and **P**^*r*^ were then standardized using the Python StandardScaler before fitting the CCA models. We computed a total of 1,000 canonical-correlations using the above sampling approach and repeated the bootstrapped CCA for each demixed source.

### 2.8 Random forest classification

We estimate the limits of using single-unit source prevalences for each unit by training random forest classifiers. Random forest classification uses several decision trees as estimators and averages the classification accuracy by averaging over the estimators. We implemented the random forest classifier from the SciKit-Learn *ensemble* library. Random forest classifiers were trained using the single-unit source prevalences as features. The training labels were one of three groupings: (1) neuron-type family, (2) discovered morpho-electrophysiological groups, or (3) excitatory-inhibitory types. For all random forest classifiers, we use five-fold cross-validation. We used the built-in grid search method hyperparameters *max_depth* and *n_estimators* which control the depth of the decision tree and the number of decision tress to average over, respectively (Supplementary Figure 2). From the grid search, we selected the parameter combination resulting in the greatest classifier performance (using the out-of-bag score). The final classification accuracy for any given label grouping was scored based on withholding 20% of the units for the test set prior to performing the grid search. All confusion matrices were based on the test set of the final classifiers. The importance of each feature was obtained from the final random forest classifier models using built-in methods. We computed the impurity-based importance (Gini importance) using the default *feature_importances_* method for RandomForestClassifier in Scikit-learn.

In order to assess the relative performance of our demixed source prevalences in classification tasks, we compare to a more traditional approach using multiple, single-channel features extracted from the center waveform and two multichannel features. The single-channel features include peak-to-trough duration, depolarization half-width, peak-to-trough ratio, repolarization slope, and recovery slope. The trough is defined as the minimum value of the waveform, and the waveform peak is the maximum value following the trough. Depolarization half-width is defined as the time difference between the waveform values corresponding to 50% of the waveform trough voltage. The repolarization and recovery slopes (μV/ms) were calculated using the value of the waveform at the trough and peak, respectively, along with the values 30 μs after. We included two multichannel features: multichannel spread and total propagation velocity. The spread is defined as the range in distance in microns across all channels with amplitudes >12% of the maximum amplitude. For the total propagation velocity, first we extract the time of the trough for all channels. We then compute the absolute value of the median slope (μm/ms) between the center channel and all channels above (propagation velocity above) and also below the center channel (propagation velocity below). Slope is defined as the distance between channels divided by the time difference of the troughs. The total propagation velocity is then taken to be the sum of the two slopes from above and below. We applied a random forest classifier as defined previously with the added constraint that *max_features* is taken to be four, the number of sources we are comparing against. This has the effect that only four randomly chosen (without replacement) features are used to construct a given random tree within the ensemble.

### 2.9 Clustering methods

To benchmark the performance of our methods, we performed standard unsupervised learning to group single-unit source prevalences into putative excitatory and inhibitory types to compare to their true class labels. We applied *k*-means and agglomerative clustering from the SciKit-Learn *cluster* library and Gaussian mixture models from the *mixture* library. In the case of agglomerative clustering, we applied both Ward's method and the average linkage method for merging the hierarchical clusters. In all cases, Euclidean distance was used as the distance metric. We also took the number of groups (either *n_clusters* or *n_components*) to be two. To assess how well each method performed on this benchmark, we computed the percent correct clustered using the the true labels.

## 3 Results

### 3.1 Unsupervised discovery of EAP spatial and multi-timescale dynamic patterns of EAP sources

Here we describe the overall demixing strategy for discovering reliable morpho-electrophysiological (ME) types across multichannel recordings of EAPs. The approach is validated using biophysical simulations from morphologically-realistic cortical neuron models. Overall, the strategy finds a reduced set of population-level spatial patterns (*spatial sources*) and multiresolution dynamics (*temporal sources*) that describe variability across simulated or recorded EAP waveforms. These *EAP sources* expose the contribution of specific morphological domains and electrophysiological phases to EAPs. Each unit can be represented by different relative contributions (*prevalences*) of these spatial and temporal sources. A major strength of this approach is its ability to uniquely represent each unit within a given dataset as a combination of EAP sources which are predictive of ground-truth morphological properties. We can also find the prevalence of these sources at different spatial locations, times, and timescales across neuron-types.

Our strategy consists of four stages. First, automated feature extraction is used to represent multiresolution dynamics of EAPs recorded on individual channels. Second, a tensor decomposition is obtained that represents each of the isolated units in terms of shared multiresolution dynamic patterns across channel locations (i.e., demixing of EAP spatial and temporal sources). Third, a supervised method demonstrates the limits of this approach for multiple parameterizations of the tensor decomposition. Finally, a latent morpho-electrophysiological space is constructed to represent neuron-types using single-unit (SU) source prevalences. We show that neuron-type representations in the morpho-electrophysiological space are correlated to local model morphometrics. We also use these representations for unsupervised clustering of neuron-types.

Our strategy emphasizes the analysis of demixed EAP sources that summarize the trial-by-trial variability across the single-unit EAP spatiotemporal waveforms (Figure 1B) without trial averaging. These demixed EAP sources describe how each single-unit differs based on shared temporal dynamics across multiple timescales and where those patterns are more localized relative to the soma. These EAP sources can be examined to generate hypotheses about how populations of latent neuron-types can be discovered from multichannel recordings. Interpretations of EAP sources are supported by correlating the single-unit source prevalences with local morphometrics to predict the morphological properties discovered through demixing EAP sources.

### 3.2 Four source model approximates multichannel EAP waveforms and predicts neuron-type families

When applying tensor components analysis (TCA), we must choose an appropriate number of components to describe the data (see Section 2.5.3). This approach discovers a low-dimensional representation of the dataset. Additionally, random initiations can lead to quantitatively different solutions. We found that there were qualitatively similar patterns across random initiations despite differences in the exact solutions. In order to constrain the demixed source model, we studied the applicability of TCA by comparing independent trainings of the model, determining a similarity score for each number of sources (or components).

We first varied the number of sources and tested four independent trainings. We found that the average similarity scores were larger than 99% for up to four EAP sources before rapidly decaying (Figure 3B). While the standard experimental setting would not have access to ground-truth information on morphological types, the use of computational models from defined neuron-type families facilitated further constraining the number of desirable EAP sources. To that end, we determined the predictive power of the demixed source models for up to four sources using a random forest classifier. Each classifier was trained to predict the neuron-type family of a simulated unit based on the single-unit source prevalences. We found that the classification accuracy steadily increased with the addition of each new source (Figure 3C).

**Figure 7:**
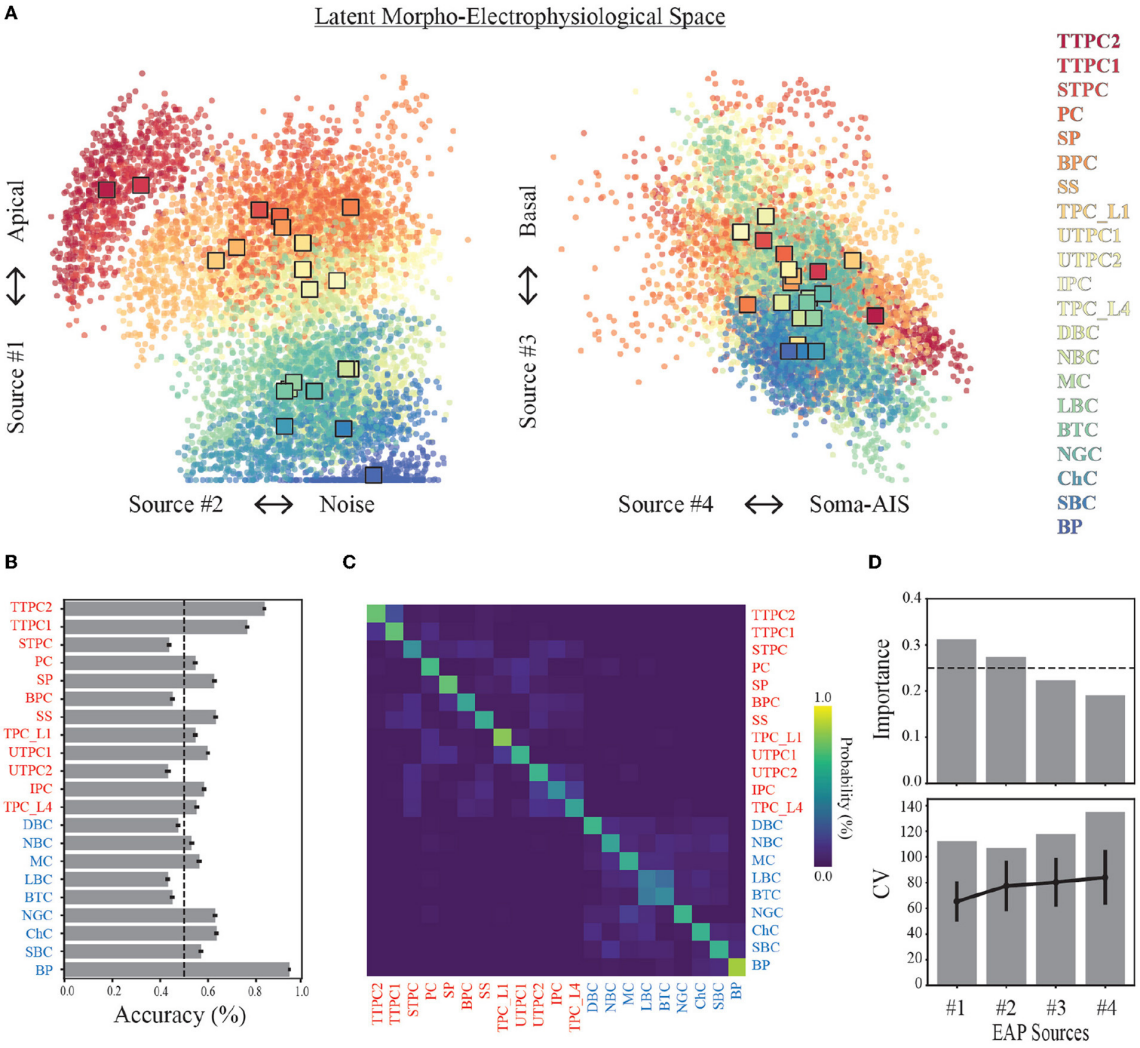
Source prevalences for units predict neuron-type model families. **(A)** Each point in the scatter plot corresponds to a unit (a model and probe location), where the color indicates the neuron-type family (color legend on the right). The location of the point corresponds to the source prevalence for the sources shown on the axes. This 4-dimensional space illustrates the latent morpho-electrophysiological (ME) space and shows the contribution of each source to recorded multichannel EAPs. The expected demixed representation of each neuron-type family (neuron-type representation, colored squares) are depicted by the bootstrapped centroids using 1,000 random subsamples. **(B)** Average prediction accuracy for random forest classifier (out-of-bag score) is shown for each neuron-type family. Dashed line depicts 50% threshold. **(C)** Confusion matrix from random forest classifier across all neuron-types. Neuron-type labels show excitatory (red) and inhibitory (blue) families. Ground truth is comprised of model neuron-types, and predictions are predicted model neuron-types. Color indicates probability of predicted neuron-type family normalized by row. **(D)** Feature importances estimated by random forest classifier using 100 random subsamples of simulated units (top). Dashed line depicts the threshold for equally weighted importances. 95% confidence intervals are too small to show. Coefficient of variation for change in prevalence with distance (bottom) across all units (gray bars) and average values for each neuron-type family (black lines, error bars are one standard deviation).

These results identify four unique sources that most contribute to spatiotemporal patterns shared across multiple neuron-types recorded on linear probes (Figure 4). Note that we ordered the EAP sources by decreasing importance of each source to classifying the neuron-type families using the random forest classifier (Figure 7D, top). By visualizing the spatial and multiresolution patterns associated with each source, we found that patterns discovered using fewer sources were qualitatively reproduced in the final four source model. This suggests that the discovered EAP sources may reflect fundamental differences across neuron-types that could be reproduced across many conditions.

### 3.3 Multiresolution temporal sources reflect different action potential phases

We proposed that the discovered four source model reflects fundamental differences across neuron-types. In order to evaluate that proposition, we investigated how the multiresolution temporal and spatial patterns of the EAP sources could be interpreted in terms of the electrophysiology and morphology of the neuron-type models, respectively. We first reconstructed the four EAP sources in terms of the multiresolution temporal patterns and interpreted the approximated dynamics of each source.

We divided the dynamics of the canonical waveform of the somatic action potential into electrophysiological phases (Figure 5A, top). We took the canonical waveform to be the average of all somatic IAPs (normalized and aligned by their peaks). The elecrophysiological phases were defined as: capacitive (I), depolarization (II), repolarization (III), and recovery (IV) phases. We then mapped these phases onto the canonical waveform of the EAPs closest to the perisomatic region (Figure 5D, bottom). We next reconstructed the dynamics by taking the product of the contributions of the discovered multiresolution patterns and Haar wavelets at the corresponding times and timescales. Since the EAP sources are all expressed as non-negative contributions, the resulting source reconstructions primarily reflect the magnitude of the contribution in time for a reconstructed temporal source. We found that the reconstructed source dynamics exposed electrophysiological phases with the most variability across neuron-type models, and that the dynamics predominately differed in the repolarization and recovery phases across all models (Figure 5B, gray regions).

More specifically, Source #1 is associated with changes in the recovery phase (centered at 2.1 ms), as well as fast changes during the capacitive phase (Figure 5B). Recall that this source was the most important source for classifying neuron-type families. The variability for all IAPs peaked during the initial part of the recovery phase at ~1.75 ms. Due to the delay between the time of peak variability of IAPs and the time of peak contribution after the spike time of Source #1, we conclude that this source is likely associated with propagation into dendrites. Further, the high contribution during the capacitive phase also points to plausible propagation of strong axial currents into large dendritic compartments (Gold et al., 2007). Together these results suggest that Source #1 may reflect active dendritic processes activated by backpropagation, similar to results found by Jia et al. (2019). This is the subject of ongoing further research.

The next most important sources were Sources #2 and #3 in that order. Source #2 is associated with increasingly rapid fluctuations starting at the beginning of the recovery phase. This source monotonically increases in the magnitude of its contribution with time. As it is least present during the active phases of the action potential and it contributes at channel locations most distal from the soma, we concluded that Source #2 reflects the constant background noise level present in all extracellular recordings. In contrast to Source #1, Source #3 is associated with the middle of the recovery phase (centered at 2.8 ms) and its contribution is observed on a slower timescale. Additionally, Source #3 peaks at greater distances (± 60 μ*m*) from the soma while also contributing the least nearby the soma. This, together with the smaller contribution from the capacitive phase, points to propagation of the action potential into more distal regions of dendrites. As these distal dendrites have weaker axial currents due to their smaller diameters, their contribution can likely only be observed when the more dominant contributions of larger dendritic compartments and the soma have dissipated.

Finally, Source #4 is associated with fast timescale differences during the repolarization phase that partially carry over into the recovery phase. This source mostly contributes at channels nearest to the soma. Unlike other EAP sources, this source retains nearly 50% of the peak spatial contribution across all channels (Figure 5B inset, Figure 4D). Because of this wide reaching contribution, the peak at the center channel, and its association with the repolarization phase, it's likely that Source #4 is largely due to differences in the somatic and AIS currents which dominate EAPs (Bakkum et al., 2019). The two additional peaks aren't interpretable at this stage of analysis. Interestingly, the variability in this phase has historically been exploited for classifying units from single-channel recordings as putative excitatory and inhibitory types by computing spike half-widths or spike durations. This variability is apparent by visual examination of the EAP waveforms at the center channel during the repolarization phase (Figure 5A, bottom). However, this was the least important source for classifying neuron-type families as previously indicated.

### 3.4 Spatial sources reflect the population density of different morphological domains

We now visit the problem of interpreting the spatial sources in terms of the morphological properties of the neuron-type models. Cortical neurons can exhibit both symmetry in morphological domains and certain deep-superficial layer biases. For example, deep layer cortical pyramidal cells often have large apical dendrites that project superficially. However, some cortical pyramidal cells exhibit apical dendrites that project toward deeper layers or even bidirectionally. Differences between EAP sources were most apparent in the patterns of the spatial sources (Figure 4, left column). Each spatial source exhibited symmetry about the center channel (soma region) in the approximate locations of their peak contributions with slight deep or superficial layer biases in the exact contribution. As evidenced by the analysis of multiresolution temporal sources, the patterns associated with each of the spatial sources may reflect differences in propagation of the AP into proximal or more distal dendrites. Excluding Source #2 associated with background noise, we asked whether the spatial sources were related to the spatial distributions of different morphological domains along the axis of the simulated probes.

We divided the neurites of the neuron-type models into three domains: AIS, basal, and non-basal/apical (Figure 6A). We computed multiple features for each these morphological domains along the axis of the simulated probes and relative to the soma center for the respective neuron-type models (Figure 6A). We first found the proximal locations of each stem (y_*prox*_), the locations of the first branch of each stem (y_*dist*_), and the locations of all terminal ends of a stem (y_*term*_). From these, we then computed for the morphological domains of each neuron-type model: the maximal extent of each domain for upper and lower divisions of the soma, the average locations of the initial branch of each stem for upper and lower divisions, and the average locations of terminal ends for upper and lower divisions. By comparing the population densities of these features for the different domains to the spatial sources, we found that the contribution of the spatial sources along the probe axis reflected different morphological domains.

As mentioned above, we hypothesized that Source #1 has a delayed contribution to EAPs that peaks during the initial part of the recovery phase. We found that the spatial contributions peaked ~40 μ*m* above and below the center channel. Source #1 is largely associated with both the average location of the first branch of apical dendrites and the average locations of apical terminals (Figure 6B). While these were plotted together to show this relationship, the upper peak (40 μ*m*) is mostly associated with the first branch location of apical trunks while the lower peak (−40 μ*m*) is mostly associated with the apical terminals. Additionally, the rising contribution near 150 μ*m* also reflected apical terminals. Alternatively, we hypothesized that Source #3 is associated with smaller compartments in the distal regions of dendrites due to its delayed contribution and relatively small contribution during the capacitive phase. Here we found that Source #3 followed a pattern similar to the spatial distribution of basal terminal ends across neuron-type models.

In the previous section, we were not able to interpret the upper and lower peaks of Source #4 based solely on the reconstructed temporal source dynamics. We found that any one single morphological domain was not able to reproduce the locations of the peaks in the spatial source. Instead, the central peak was associated with the combined distribution of average initial branch locations for all stem domains. Alternatively, the upper and lower peaks located at 120 and −100 μ*m*, respectively, followed the upper and lower maximum extent of basal dendrites. Note that the basal dendrites were often present at farther distances from the soma than the spatial source would suggest. It's likely that the background noise overcame the contribution of the most distal basal terminal ends. Further, the average terminal ends of basal dendrites of Source #3 correspond to the lowest points of the Source #4, suggesting a different underlying mechanism. Together these results suggest that the two additional peaks correspond to current sinks associated with the current source during the repolarization phase of the somatic action potential as opposed to the contribution of active dendritic processes to the EAP.

### 3.5 Single-unit source prevalences predict local morphological properties

We demonstrated plausible underpinnings of the demixed EAP sources by examining the relationship between temporal sources and electrophysiological phases of the action potential, as well as between spatial sources and spatial distributions of morphological properties. These EAP sources contain information about the total population of neuron-type models. What remains to be seen is how the variability in these population-level patterns at the individual unit level is reflected in the single-unit source prevalences. Are the associated single-unit source prevalences predictive of biases in morphology?

To address the question of whether we can identify key biases in morphology from source prevalences, we performed a bootstrapped canonical correlation analysis (CCA). We estimated the cross-covariance matrix between the local morphometrics across the neuron-type models and 100 single-unit source prevalences randomly sampled with replacement from the corresponding neuron-type models and repeated this process 1,000 times. This enabled discovery of the canonical co-variate that describe the relationship between these two representations of the neuron-type models. By computing the correlation coefficient (Pearson's R) for the local morphometrics and canonical covariate, we estimated how well the single-unit source prevalences predict local morphometrics. We found that overall the single-unit source prevalences predominately vary with asymmetries in neurite CSAs (Figure 6C).

Bootstrapped CCA showed that neurons with more apical terminals had larger contributions from Source #1, congruent with previous analyses. Additionally, these neuron-types also had axons with larger CSAs that projected downward. While the total CSA of apical stems varied with the prevalence of this source, it also varied more if that stem was superficially oriented. The inverse relationship between the soma height along the probe axis and the prevalence of Source #1 seems to suggest that there is a trade off between the stem geometry above and the size of the soma. Source #2 appeared to be inversely related to all stem CSAs with some biases in the upper and lower division. This was expected as the total stem CSA is proportional to the maximum amplitude of EAPs (Pettersen and Einevoll, 2008), and we associated Source #2 with the background noise level. Interestingly, neurons with larger basal stem CSAs were inversely related to Source #3 while larger stem CSAs in the upper division varied with the source prevalence. This upper-lower bias is reflected in the profile of its spatial source. This may be explained by the fact that neurons with basal dendrites also tended to have larger non-basal/apical dendrites, resulting in more contribution from Source #1 than Source #3 as their corresponding peaks in spatial prevalences occupied similar channel regions. Finally, Source #4 was largely correlated with increasing soma and axon sizes, but also positively varied with the total CSA of all other neurites as well.

### 3.6 Single-unit source prevalences differentially scale with recording distance

For the final interpretive analysis, we asked whether neuron-type specific differences in source prevalences predictably vary with recording distance and do these variations support our predictions about the underlying electrophysiology? Our previous analyses implicated specific source-morphological domain relationships. Source #1 was associated with the apical domain, Source #2 with background noise, Source #3 with the basal domain, and Source #4 with the soma-AIS domains (Figure 6D). Given the predicted morphological domains contributing to each EAP source, we found that the single-unit source prevalences scaled with distance mostly as expected.

Recall that each neuron-type family consists of five morphological variants. Here we grouped all single-unit source prevalences within their respective families. From there, we took the source prevalence of the unit nearest to its corresponding neuron-type model within a family and computed the differences in source prevalence of all other units relative to the source prevalence of the nearest unit (Figure 6D). The prevalence of Sources #1 and #3 associated with dendritic domains both increased with recording distance. In contrast, the prevalence of Source #4, associated with the soma-AIS domains, decreased with distance. It is known that the contribution of somatic currents to EAPs decays with increasing recording distance while the contribution from dendritic currents increases with distance (Buzsáki and Kandel, 1998; Pettersen and Einevoll, 2008). This is largely due to the low-pass filtering properties of dendrites and the extracellular space (Bédard et al., 2004; Pettersen and Einevoll, 2008). Since the contribution of the soma to EAPs decays with distance, the background noise level should increase with distance, as well. As expected, Source #2 associated with the background noise increased with recording distance.

Our analysis also revealed that the prevalence of the contribution from the apical domain scales more strongly with distance compared to the basal domain. Additionally, the slope of the prevalence scaling of the background noise and the basal domain contributions were comparable. We previously noted that the spatial distribution in the basal domain peaked at greater distances than the spatial profile of Source #4 (Figure 6B). The comparable scaling in prevalence of Sources #2 and #3 supports that the noise level eventually overcomes any contribution from the basal domain at more distal recording sites along the probe axis. This is not immediately observed by examining Source #4 in isolation.

### 3.7 Neuron-types primarily differ in the prevalence of apical and noise sources

We now examine the problem of identifying neuron-type families using the single-unit source prevalences. We constructed a latent morpho-electrophysiological space using the source prevalences. The main goal in constructing this space is to determine the expected representation of neuron-type families under the four source model. Additionally, we aim to determine the robustness and accuracy of these neuron-type representations while defining a hierarchy of morpho-electrophysiological types based on their similarity within this space.

Each neuron-type family was represented by the corresponding centroid of the single-unit source prevalences (Figure 7A). We used bootstrapping to compute median centroids using 1,000 datasets where each dataset consisted of 80% of the total dataset obtained by random sampling with replacement. Three broad classes of neuron-type families corresponding to thick-tufted pyramidal cells (red), all other excitatory cells (orange-yellow), and inhibitory cells (green-blue) can be visually identified within this space using only Source #1 and #2. This further illustrates a trade-off between the contributions from the apical domain and background noise. Thick-tufted pyramidal cells have the largest apical trunks and are well isolated within this space. While many of the other excitatory families have apical dendrites (orange-yellow), they have noise levels that are comparable to the interneuron families (green-blue).

We examined the limits of this approach for identifying the neuron-type families by training a random forest classifier to predict the neuron-type family from the single-unit representations in the morpho-electrophysiological space. We found that the average classification accuracy across morphological types was ~60% (Figure 7B). Thick-tufted pyramidal cells (TTPC) and bipolar interneurons (BP) were the most likely to be correctly classified while six neuron-type families could not be identified above 50%. However, the majority of misclassifications occurred within excitatory and inhibitory groups as evidenced by the confusion matrix (Figure 7C). The importance of each source for classifying neuron-type families supported that Sources #1 and #2 best predict neuron-types from single-unit source prevalences based on the random forest classifier (Figure 7D, top). Further, we computed the variability in prevalence for each source and compared that to the neuron-type specific variability for each source and found that neuron-type families were well separated with respect to the morpho-electrophysiological space (Figure 7D, bottom).

### 3.8 The four EAP source model defines a hierarchy of neuron-types robust to multiple sampling biases

In the previous section, we found that the single-unit representations within morpho-electrophyiological space were sufficiently separated to classify individual neuron-type families 60% of the time on average. We took this to be the lower limit of our neuron-type identification strategy based on EAP waveforms. We constructed a hierarchy of latent morpho-electrophysiological (ME) types, where our goal was to establish broad classes of neuron-type families with similar representations in the constructed space that could serve as a plausible multi-level classification scheme.

**Figure 8:**
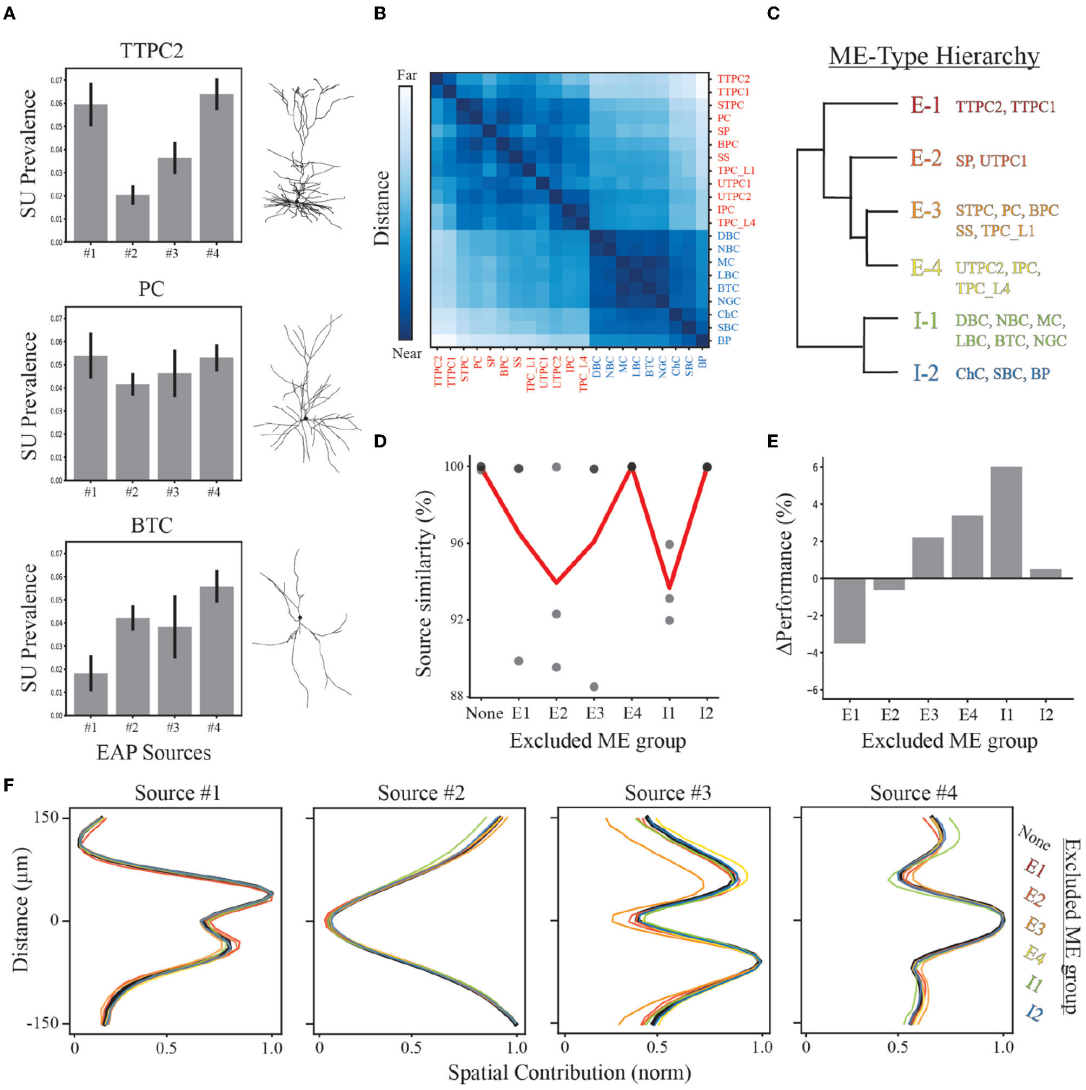
Demixing sensitivity to exclusion of neuron-type families. **(A)** Example neuron-type source prevalence representations for thick-tufted pyramidal cell (TTPC), pyramidal cell (PC), and bitufted cell (BTC) families with example morphologies. Error bars depict one standard deviation. **(B)** Distance matrix for neuron-type representations based on source prevalence centroids for each neuron-type family shown in Figure 7A. Excitatory and inhibitory populations are labeled in red and blue, respectively. Matrix color values range from 0 to 1, representing the normalized Euclidean distance between these centroids in feature space. **(C)** Reduced dendrogram based on neuron-type representations illustrates morpho-electrophysiological (ME) hierarchy. **(D)** Multiple demixed characterizations are learned for different combinations of excluded neuron-type families based on ME hierarchy and their similarity scores (black circles). The average similarity (red line) depicts how excluding different ME-types impacts the overall quality of the demixing. **(E)** The performance of random forest classifiers are compared for each exclusion condition relative to the original demixing. **(F)** Resulting spatial source contributions for all exclusion conditions (colored lines) and the original demixing (black line) demonstrating robust qualitative correspondences in EAP sources.

The hierarchy was defined by computing a distance matrix for all neuron-type representations (Figures 8A, B). We assigned the neuron-type representations based on the distance matrix between bootstrapped centroids. We chose a distance threshold for assigning the families to ME-types that yielded a hierarchy which contained six broad classes of neuron-type families that were most similar to each other within morpho-electrophysiological space (Figure 8C; Supplementary Figure 3A). As expected, excitatory and inhibitory representations are consistently closer to other members within their respective EI class (Figures 8B, C). While excitatory families make up ~57% of the total neuron-type families used here, the hierarchy illustrates that twice as many excitatory classes can be determined from EAP waveforms than inhibitory classes.

We performed a “leave-one-out” analysis that excluded a specific ME-type and performed the demixing analysis on data from the remaining types. As each ME-type is defined based on neuron-type families with similar representations, this analysis accounts for sampling biases that would exclude similar EAPs and could potentially impact our results as early as the recording step. Discovered EAP sources exhibited average similarity scores >92% despite the exclusion of different ME groups (Figure 8D). We then assessed the ME-type hierarchy as a multi-level classification scheme using random forest classifiers. We found that all units could be correctly classified as an ME-type ~70% of the time on average (Figure 7E; Supplementary Figures 3B, C). This was roughly a 10% improvement above classifying neuron-type families. Additionally, excluding any ME-type from the entire pipeline resulted in only small changes in classification accuracy (approximately −4 to 6%, Figures 8E, F). A decline in classification accuracy was observed only from exclusion of thick-tufted pyramids (E1), and star pyramids and untufted pyramids (E2). In the remaining cases, classification accuracy actually improved by as much as 6% (when excluding I1-type neuron-types, Figures 8E, F).

Since the contributions from the four spatial sources were the easiest to qualitatively compare, we examined the qualitative correspondence of the spatial sources discovered from the exclusion datasets. We found that the peak location of each of the sources was the same across all exclusion conditions and the overall shape was nearly identical. The greatest difference across all exclusion conditions was seen in Source #3 when the E3 group was removed. Despite this ME group not having the largest source prevalences for Source #3, the exclusion of the E3 group resulted in a decrease in the upper peak of Source #3 indicating that E3 was the largest source of variability within that region. Overall, the consistent qualitative correspondence of EAP sources across all conditions point to our demixing strategy being robust under many different conditions.

### 3.9 Demixed representations enhance model selection and accuracy for neuron-type identification

**Figure 9:**
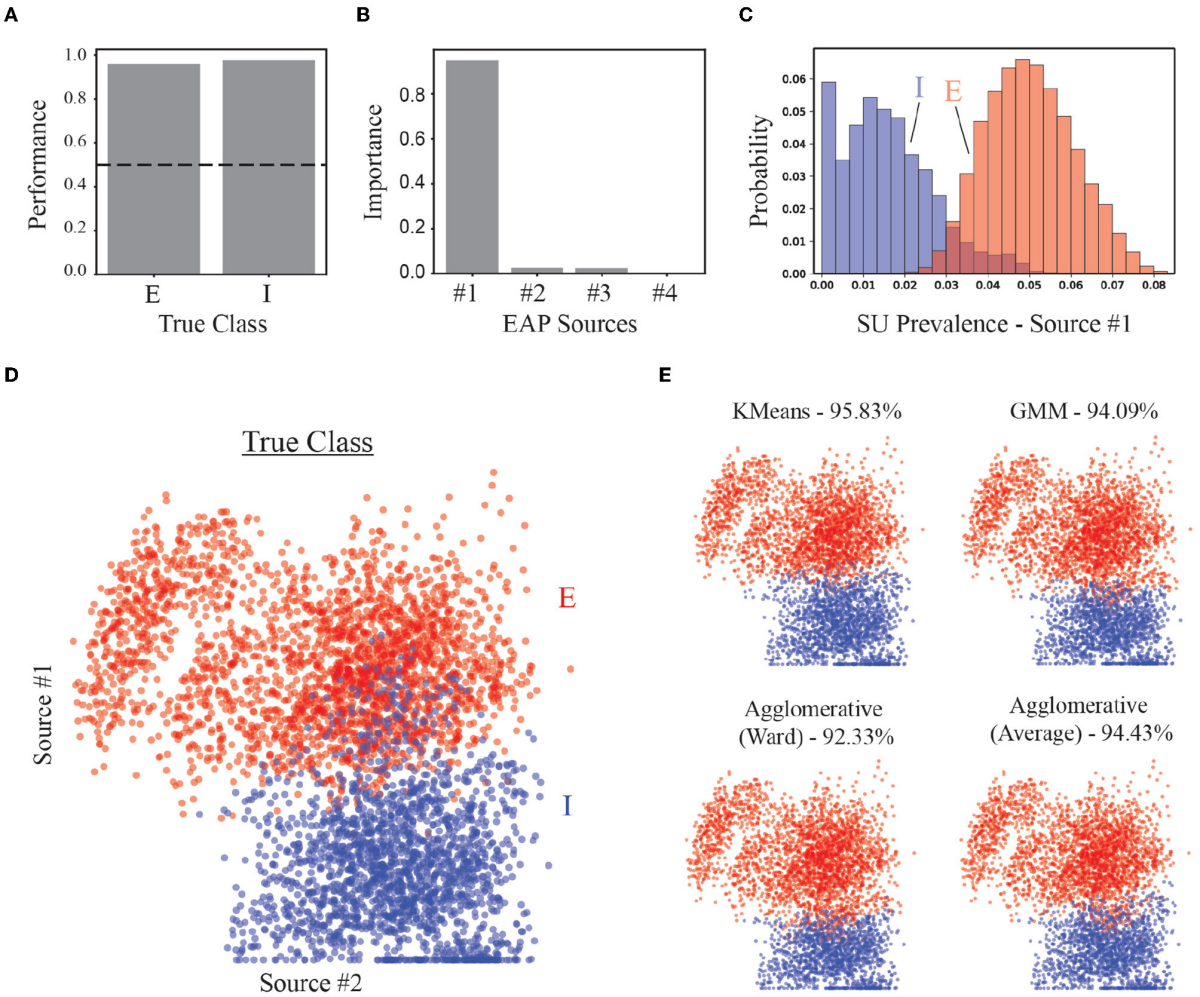
Performance of EI classification based on source prevalences. **(A)** Classification accuracy of random forest classifier for excitatory (E) and inhibitory (I) populations. Dashed line shows 50% accuracy. **(B)** Feature importance for random forest classifier. **(C)** Histogram of most important feature (Source #1) for distinguishing E vs. I simulated units. **(D)** The location of each point in the scatter plot represents the source prevalences for the first two EAP sources. The color of the point depicts the actual EI class for the corresponding unit. **(E)** Results from implementation of multiple unsupervised methods used to discover two groups corresponding to E- and I-types. Percentages are the average true positive rates.

As a benchmark for the performance of our strategy, we assessed the accuracy of our approach in identifying putative excitatory and inhibitory units in both supervised and unsupervised contexts. A random forest classifier trained to classify excitatory and inhibitory classes demonstrated high accuracy (~96%) using predominately the prevalence of Source #1 (Figures 9A, B). Recall that Source #1 reflects the contribution of apical dendrites to the initial recovery phase of EAP waveforms surrounding the soma region (−40 to 40 μ*m*). The prevalence of this source was largely absent from units of inhibitory families (Figure 9C). The non-zero source prevalence may reflect inhibitory types with basal dendrites that have larger CSAs which could be termed apical-like, i.e., elongated basal dendrites with delayed branching that were more aligned with the probe axis. We also compared how well different clustering methods could separate excitatory and inhibitory neurons using the source prevalences (Figure 9D). All methods could correctly identify more than 90% true EI classes for the units (Figure 9E).

Finally, we assessed the performance of the four source model compared to a more traditional approach using engineered features (see Section 2). We trained random forest classifiers for each of the classifications (EI-types, ME-types, and neuron-types) and consider this as a baseline for classification performance. Comparisons between four source and baseline classifications were based on model accuracy and the number of relatively important features. We found that the performance of the four source model exhibited comparable performance to the baseline classifiers for both EI types (97 and 98%, respectively) and ME types (83 and 81%, respectively). However, baseline classifiers required a larger number of features to attain comparable performance (indicated by relative feature importances as shown in Supplementary Figures 4A–C, left).

For EI classification, the ratio of important features of the four source classifier to baseline classifier was 1:2 (Figure 9B; Supplementary Figure 4A). Here the repolarization and recovery slopes contributed the most to classifier performance as opposed to more traditional features used such as waveform duration or half-width. For ME-type classification, the ratio of important features was 1:4 (Supplementary Figures 3C, 4B). While the ME classes were defined within the latent ME space of the four source model, potentially exaggerating this difference, this result suggests that the prevalence of the discovered apical source may also contribute to variability in waveform duration.

The most significant difference in performance for the baseline features vs. the four source features was for classifying neuron-type families. The ratio of important features was 2:3 (Figure 7D; Supplementary Figure 4C). While not a dramatic difference in the number of important features, the baseline performance was 58% for classifying neuron-type families compared to 63% for the four source model (Supplementary Figure 4C; Figure 6B). Together these results suggest the most significant benefit of our approach is the unsupervised discovery of novel features for enhanced identification of neuron-types.

## 4 Discussion

We demonstrate a machine learning approach for the unsupervised discovery of features for neuron-type identification. Our goal was to find differentiating patterns in EAP waveforms across space that span multiple timescales. To that end, our approach adopted a demixing strategy consisting of two stages: (1) multiresolution waveform representation and (2) tensor decomposition. We included models from a diverse set of cortical neuron-type families and performed forward modeling of EAPs for more than 100 morphological variants. One advantage of our study was the incorporation of empirically-based conditions for background noise. Another feature was the implementation of detectability ranges caused by the attenuation of extracellular potentials with distance, consistent with experimental observations. Combining these two aspects enhanced the heterogeneity of our training dataset and generalized our results beyond previous modeling studies.

Our approach discovered features that reflect the population-level prevalence of EAP sources that relate to morphological domains across neuron-types. We identified four unique EAP sources present across spatiotemporal EAP waveforms that could be robustly discovered across multiple conditions. These EAP sources had differentiating contributions to the EAP waveform during specific phases of the action potential, and changes in their contribution with distance yielded results in agreement with previous experimental and modeling studies. These results further characterized the complex relationship between EAP sources, neuron-types, and recording distance for each individual multichannel waveforms, allowing for enhanced classification of recordings. Thus, our modeling study illustrates the applicability of this demixing strategy for neuron-type identification, as well as the plausibility of neuron-type identification from high-density extracellular recordings.

### 4.1 Validation of machine learning methods with computational modeling

Previous studies have used computational models of cortical networks and microcircuits to validate conventional data analytic techniques such as independent components analysis (Głbska et al., 2014), spike sorting (Hagen et al., 2015), current source density analysis (Łeski et al., 2007, 2011; Ness et al., 2015), and multi-linear population analysis (Geddes et al., 2020). These model-based studies of data analysis techniques are not limited to conventional data analysis techniques, but can be applied to modern techniques for parameter estimation for the further development of both simulation-based inference and development of mechanistic models (Einevoll et al., 2007; Gold et al., 2007; Głbska et al., 2016; Buccino et al., 2018; Goncalves and Lueckmann, 2020; Haynes, 2020; Skaar et al., 2020; Tejero-Cantero et al., 2020; Birgiolas et al., 2023). In the present study, we focus on validating a machine learning strategy using tensor components analysis within a new empirical context - the identification of the underlying sources that contribute to patterns within spatiotemporal EAP waveforms and the subsequent identification of neuron-types. Additionally, previous model-based studies that focus on classifying neuron-types based on EAPs neglect the influence of background noise and detectability (Somogyvári et al., 2005, 2012; Delgado Ruz and Schultz, 2014; Buccino et al., 2018). Empirical decay constants of EAP amplitude have been estimated to be ~28 μ*m* (Gray et al., 1995; Segev et al., 2004). As this signal attenuation results in more contamination by noise sources, an advantage of our study is the incorporation of these biophysical constraints thus including boundary conditions for the extracellular space of our simulated datasets.

### 4.2 Correspondence of EAP source prevalences to waveform features

The four demixed EAP sources were comprised of: an apical source (Source #1), a noise source (Source #2), a basal source (Source #3), and a soma-AIS source (Source #4). Each source was characterized by the spatial distribution of the compartments associated with the respective morphological domains, dynamic signatures during different electrophysiological phases, their prevalence across the population of neuron-type families, and the tendency to either increase or decrease in prevalence with recording distance. These four EAP sources are summarized in Supplementary Table 2.

As demonstrated above, the tensor of spatiotemporal EAP waveforms, **X**, is generated by several morphological sources and also contaminated by an additive noise source. By systematically varying R and visualizing the R EAP sources, we find that the noise source is present across all values of R. Interestingly, this background noise source was more important in differentiating neuron-types at the level of neuron-type families than both the basal and soma/AIS sources. These results suggest that characterizing the change in noise-related features across the channels and during the the last phase of the EAP waveforms would be an effective feature for neuron-type classification. One example of this approach uses waveform amplitude spread for classifying units according to visual cortical area based on Neuropixel probe recordings (Jia et al., 2019). Of the features considered in the study, the amplitude spread was the least important for classifying brain areas. However, by clustering units into fast spiking (FS) cells and two types of regular spiking cells (RS1 and RS2), they found that FS cells had significantly broader amplitude spread than RS2 cells and comparable spread to RS1 cells. Further, RS1 cells had significantly broader spread than RS2. The prevalence of the noise source as discovered by our study yielded similar results, with the inhibitory families having larger noise source prevalence than E1-types (TTPC1, TTPC2) but comparable noise source prevalence with regard to the remaining excitatory families (Figure 7A; Supplemental Figure 5).

We find that the most important feature for neuron-type identification is the prevalence of the apical source, which has the strongest contribution during the early recovery phase and the capacitive phase (see Source #1 in Figure 5B). Using paired juxtacellular and high-density extracellular recordings, Marques-Smith et al. (2018) showed that triphasic waveforms with a positive-negative-positive peak progression are consistently located above the channels closest to the soma for some neurons and are absent for others. This initial positive peak is associated with the contribution of capacitive currents to the EAP (Gold et al., 2007). This initial peak can be observed for both propagation of axial currents into the soma from the AIS (Teleńczuk et al., 2018; Bakkum et al., 2019) as well as propagation into an apical dendrite from the soma (Gold et al., 2007; Haynes, 2020) during the action potential. An important caveat is that shifting the waveforms by their median values may amplify positive biases within the pre-spike phase; however, changes in this median value across channels could be used as a feature to describe the contribution of the apical source.

Further, we find that the spatial contributions of the apical source reflect the locations of the stems of the apical dendrites and any nearby apical terminals. The prominent bias toward the channels above the center channel and the delayed contribution to the EAP waveform during the recovery phase suggest an overall bias in upward propagation after AP initiation (see Figure 6). It has been shown that the time between the positive peak after the EAP waveform trough and the next inflection point during the recovery phase aids in differentiating multiple functional classes of units from single channel recordings (Trainito et al., 2019). These units tended to have center channel EAPs of longer duration, which is typically associated with pyramidal cells, in agreement with the more prominent contribution of the apical source to the pyramidal neuron-type families.

By computing the propagation velocity above and below the center channel using EAP waveform troughs for RS cells, Jia et al. (2019) found a similar slow upward propagation of EAPs relative to FS cells. This propagation extended farther for RS1 cells than RS2 cells. Moreover, RS2 and FS cells exhibited slow and fast symmetric propagation, respectively. Given the contributions of the basal source (see Source #3 in Figures 5B, 6B), which peaks during the middle of the recovery phase, this source may correspond to symmetric propagation away from the soma. In Jia et al. (2019), FS cells had fast, symmetric propagation, while RS2 cells had slow, symmetric propagation. It's plausible that the RS1 cells correspond to the E1-types in our study with the RS1 and FS cells corresponding to the remaining excitatory and inhibitory families, respectively.

Finally, the soma/AIS source contributed most at the center channel and was associated with the late repolarization phase (see Source #4 in Figures 5B, 6B). This likely corresponds to the standard approaches for differentiating excitatory and inhibitory cells by using the spike half-width or spike duration. However, there was no consistent split between excitatory and inhibitory neuron models using the prevalence of this source. It was also the least important source for classifying inhibitory and excitatory types as determined by the random forest classifier. This was likely due to the mixed contributions from other neurites for this source.

### 4.3 Neuron-type identification: Outlook and limitations

In this study, we framed the problem of discovering features for neuron-type classification and identification as EAP source demixing. Historically, the problem of spike sorting is framed as source separation (Oweiss and Aghagolzadeh, 2010) and the standard approach to this uses principal components analysis (PCA) to represent units by a reduced set of latent features. In demixing EAP sources, we identify the presence of underlying morphological sources that describe the model neuron-types using a generalization of PCA, tensor components analysis (TCA, Williams et al., 2018). We showed that a four source model best described patterns in spatiotemporal EAP waveforms discovered by TCA. Within our framework, the extracellularly recorded spatiotemporal EAP is considered to consist of multiple signal sources (morphological compartments) that are detected by a set of sensors (channels) and observed as a superimposed, mixed signal (EAP waveforms). Within an experimental setting, the exact noisy mixing process is unknown since the specific morphology of a detected neuron, as well as, the spatial relationship between the channels and the neuron are typically unknown. The present study addresses this problem by simulating the mixing process using forward modeling of EAPs for diverse neuron-type models and including spatially distributed linear probes for each model.

Extracellular potentials have been approximated using models of different gross current and potential source geometries including: monopolar/point sources, line sources, dipolar sources, quadrupolar and higher order source geometries (Somogyvári et al., 2005; Pettersen and Einevoll, 2008; Lindén et al., 2011; Mechler and Victor, 2012; Hagen et al., 2018; Næss et al., 2021). Due to the low-pass filtering of the extracellular space, how appropriate these source-based models are to modeling extracellular potentials depends on the relative distance to the recording site to the underlying sources (Pettersen and Einevoll, 2008). Here, we used morphologically-detailed models with current sources being approximated by line sources (Lindén et al., 2014). Since the mixing process is morphology-specific and varies with recording location, we exploited this to infer differences in morphology discoverable across many units from diverse model neuron-types with multiple morphological variants. These differences were described by features corresponding to the prevalence of four different EAP sources within individual mixed EAP waveforms.

The discovered features, or source prevalences, reflect the population-level prevalence of EAP sources that relate to differences in morphological domains across neuron-types (Figure 6C). By visually comparing the source-based models found in Somogyvári et al. (2005) to our demixed four source model, the four discovered sources closely correspond to the absolute value of monopolar (Source #2), dipolar (Source #1 and #3), and quadrupolar (Source #4) source models (Figure 4, left column). The conclusions of our analysis of the four source model presented here is further supported by the correspondence between morphological domains, their stem arrangement relative to the soma, and these different source-based models (Pettersen and Einevoll, 2008). Moreover, Pettersen and Einevoll (2008) found that dipolar and quadrupolar contributions dominate at different distances from the soma. There, quadrupolar contributions peaked between 60 and 100 μ*m* and dipolar contributions dominating at farther distances as these sources decay more slowly compared to quadrupolar sources. They also predicted that EAPs have mixed contributions from both dipolar and quadrupolar sources nearer to the soma. Our results were in rough agreement with these for the shorter range of distances shown here (Figure 6D). Our study provides the first account of how putative quadrupolar and dipolar sources in close proximity of neuron models depend on neuron-type Thus we propose that our strategy exposes the population patterns in monopolar, dipolar, and quadrupolar EAP sources.

Cortical pyramidal cells primarily differ in their dendritic properties (Spruston, 2008; Kanari et al., 2019) as opposed to cortical interneurons which differ based on axonal properties (Markram et al., 2004; Jiang et al., 2015). Layer 5 thick-tufted pyramidal cells having particularly polarized dendrites and the largest apical dendrites (Ramaswamy et al., 2015). Across all levels of our analysis (neuron-types, ME-types, and EI-types), we found that the apical source prevalence was most important for identifying neuron-types. Additionally, its importance scaled inversely with the number of groups we assigned the population of units to: >80% for EI-types (Figure 9B), >50% for ME-types (Supplementary Figure 5C), and >30% for neuron-type families (Figure 7D). Despite interneurons not having apical dendrites, the two inhibitory ME-types were still most differentiated by the prevalence of this source (Supplementary Figures 5A, 6) possibly due to the putative dipolar nature of the source. Further, inhibitory ME-types were identified with higher accuracy than two of the excitatory ME-types (E2 and E4, Supplementary Figure 5B) supporting this putative dipolar source having a different origin than the apical dendrite and this difference is discovered by the demixing strategy. It's plausible that if we extended the multi-level classification scheme to include multiple demixing stages in an iterative approach, the differences between apical sources of the two excitatory ME-types would also be discovered.

The inclusion of only four sources enabled visual comparison of the spatial and multiresolution temporal sources across several conditions which supported interpretations. Moreover, the similarity scores for the four source model were still above 98% (Figure 3B). Even though the present study focused on analysis of the four source model, we found that patterns were similar enough to allow for qualitative correspondences when we included even more sources. However, by including more sources, we lost previous sources as more asymmetric sources emerged—excluding the noise source (results not shown). These sources would be useful for neuron-type identification as asymmetries in spatiotemporal waveform features such as the difference in propagation velocity above and below the soma was able to differentiate the two types of RS-types found by Jia et al. (2019). Further, these asymmetric EAP sources had peak spatial contributions that closely corresponded to the peaks of the more symmetric sources of the four source model that were lost. This indicates that asymmetries in morphological domains across neuron-types are also discoverable by our strategy while maintaining agreement with the results of this study. A more systematic analysis of a greater number of EAP sources could yield even better classification results. For example, choosing *R* to be greater than the number of channels *S* used could yield finer approximations of the underlying neuron morphologies in terms of their source-based contributions while still separating distinct morphological domains. Additionally, a quantitative comparison of simulated EAPs from morphologically-detailed neuron models to simulated EAPs using a multipolar source model formalism (Somogyvári et al., 2005; Pettersen and Einevoll, 2008) informed by the relative source contributions predicted from the four source model would further improve the results presented in this study.

While our approach can discover multichannel waveform features from experimental datasets in an unsupervised manner, our study also provides a beneficial simulation-based framework for engineering novel features for analyzing multichannel recordings and identifying their morphological and electrophysiological correlates. For example, with only four sources we see that basal and apical domains exhibit separable contributions across channels and timescales. One could differentiate proximal vs distal propagation velocity and investigate their correlations to the basal and apical source prevalence in models (similar to the analysis of morphometrics above). In our study, we used the total propagation velocity so as not to bias the baseline classifier to extra information about the asymmetries in the multichannel waveforms. While such asymmetries are warranted, the sources reported here are symmetric. However, we observed that the apical source would differentiate into upper and lower apical sources that reflect such asymmetries when using more than four sources (not shown).

Overall this modeling study illustrates the applicability of a demixing strategy for neuron-type identification, as well as the plausibility of neuron-type identification from high-density extracellular recordings. Here, we demonstrate an analysis pipeline and its interpretation that relies on having both diverse computational models and experimental recordings from the same system. Further investigation is needed to determine how the sources discovered with this approach would differ across species and brain region. Additionally, the focus of this work is on single neurons; future work will focus on complex patterns of synaptic input and the impact of nearby cells.

### 4.4 Resource Identification Initiative

We acknowledge the use of resources that are part of the Resource Identification Initiative. These include Neocortical Microcircuit Portal (RRID:SCR_022032), NeuroML Database (RRID:SCR_013705), NetPyNE (RRID:SCR_014758), NEURON (RRID:SCR_005393), Scikit-Learn (RRID:SCR_002577), NumPy (RRID:SCR_008633), SciPy (RRID:SCR_008058), and Pandas (RRID:SCR_018214).

## Data availability statement

The datasets presented in this study can be found in online repositories. The names of the repository/repositories and accession number(s) can be found below: https://github.com/vrhaynes/Haynes2023_EAPs.

## Author contributions

VH: Conceptualization, Methodology, Formal analysis, Investigation, Software, Validation, Visualization, Writing – original draft. YZ: Conceptualization, Methodology, Funding acquisition, Writing – review & editing. SC: Conceptualization, Funding acquisition, Methodology, Supervision, Writing – review & editing.
