# Supplementary material for: Discovering optimal features for neuron-type identification from extracellular recordings
Source: Front Neuroinform. 2024 Feb 2;18:1303993. doi: 10.3389/fninf.2024.1303993 (PMC10869512; doi:10.3389/fninf.2024.1303993)
Supplement: Supplementary file 1 [file Data_Sheet_1.pdf]

Supplementary Material

Families	Neuron Type	NMLDB_ID
TTPC1	Layer 5 thick-tufted pyramidal cell with late bifurcating apical tuft	NMLCL000687, NMLCL000688, NMLCL000689, NMLCL000690, NMLCL000691
TTPC2	Layer 5 thick-tufted pyramidal cell with early bifurcating apical tuft	NMLCL000695, NMLCL000692, NMLCL000693, NMLCL000694, NMLCL000696
UTPC1	Layer 5 untufted pyramidal cell	NMLCL000698, NMLCL000699, NMLCL000701, NMLCL000700, NMLCL000697
UTPC2	Layer 6 untufted pyramidal cell	NMLCL000680, NMLCL000677, NMLCL000681, NMLCL000678, NMLCL000679
STPC	Layer 5 slender tufted pyramidal cell	NMLCL000684, NMLCL000685, NMLCL000682, NMLCL000686, NMLCL000683
TPC_L4	Layer 6 tufted pyramidal cell terminating in layer 4	NMLCL000672, NMLCL000676, NMLCL000673, NMLCL000674, NMLCL000675
TPC_L1	Layer 6 tufted pyramidal cell terminating in layer 1	NMLCL000669, NMLCL000670, NMLCL000667, NMLCL000671, NMLCL000668
IPC	Layer 6 inverted pyramidal cell	NMLCL000662, NMLCL000666, NMLCL000663, NMLCL000664, NMLCL000665
BPC	Layer 6 bipolar pyramidal cell	NMLCL000659, NMLCL000660, NMLCL000657, NMLCL000661, NMLCL000658
SS	Layer 4 spiny stellate	NMLCL000652, NMLCL000653, NMLCL000654, NMLCL000655, NMLCL000656
SP	Layer 4 star pyramidal cell	NMLCL000647, NMLCL000648, NMLCL000649, NMLCL000650, NMLCL000651
PC	Layer 2/3 pyramidal cell	NMLCL000637, NMLCL000638, NMLCL000639, NMLCL000640, NMLCL000641
NBC	Layer 2/3 nested basket cell	NMLCL000494, NMLCL000492, NMLCL000496, NMLCL000495, NMLCL000493
MC	Layer 2/3 martinotti cell	NMLCL000487, NMLCL000491, NMLCL000488, NMLCL000489, NMLCL000490
BTC	Layer 2/3 bitufted cell	NMLCL000469, NMLCL000487, NMLCL000491, NMLCL000488, NMLCL000490
DBC	Layer 2/3 double bouquet cell	NMLCL000480, NMLCL000479, NMLCL000477, NMLCL000481, NMLCL000478
BP	Layer 2/3 bipolar cell	NMLCL000462, NMLCL000466, NMLCL000463, NMLCL000464, NMLCL000465
LBC	Layer 2/3 large basket cell	NMLCL000484, NMLCL000485, NMLCL000482, NMLCL000486, NMLCL000483
SBC	Layer 2/3 small basket cell	NMLCL000505, NMLCL000502, NMLCL000506, NMLCL000503, NMLCL000504
ChC	Layer 2/3 chandelier cell	NMLCL000473, NMLCL000474, NMLCL000476, NMLCL000472, NMLCL000475
NGC	Layer 2/3 neurogliaform-like cell	NMLCL000501, NMLCL000499, NMLCL000498, NMLCL000497, NMLCL000500

Table S1. **Neuron-type model identifiers from NeuroML-Database.** All neuron-type models used in simulations and analyses are grouped by neuron-type family. Each family corresponds to 5 morphological variants searchable at NeuroML-DB.org using the database identifier, NMLDB_ID.

Source	Morpho Domains	Ephys Phases	Neuron Types
#1	Apical<br>(40, -40, and 150 um)	Capacitive/Recovery<br>(early, 2.8 ms)	TTPC2 (E1), TTPC1 (E1),<br>STPC (E3), PC (E3),
#2	Noise<br>(-150 and 150 um)	Recovery<br>(late, 2.8 ms)	BP (I2), SP (E2),<br>DBC (I1), NBC (I1),
#3	Basal<br>(60 and -60 um)	Recovery<br>(mid, 2.8 ms)	TTPC2 (E1), TPC_L1 (E3),<br>NGC (I1), TTPC1 (E1),
#4	Soma-AIS\Mixed<br>(0, 120, and -10 um)	Repolarization<br>(late, 1.4 ms)	TPC_L4 (E4), IPC (E4),<br>STPC (E3), PC (E3)

Table S2. **Qualitative summary of four-source model.** Discovered EAP sources are labeled by corresponding morphological domain, electrophysiological phase, and top 4 neuron-type families. The top 4 neuron-types are based on the median values of the corresponding source prevalences. Peaks in each of the spatial sources are provided for morphological domains. The interval with the largest contribution and the associated timescale in milliseconds are provided for electrophysiological phases. The morpho-electrophysiological type to which each neuron-type family belongs to is provided for top 4 neuron-type families.

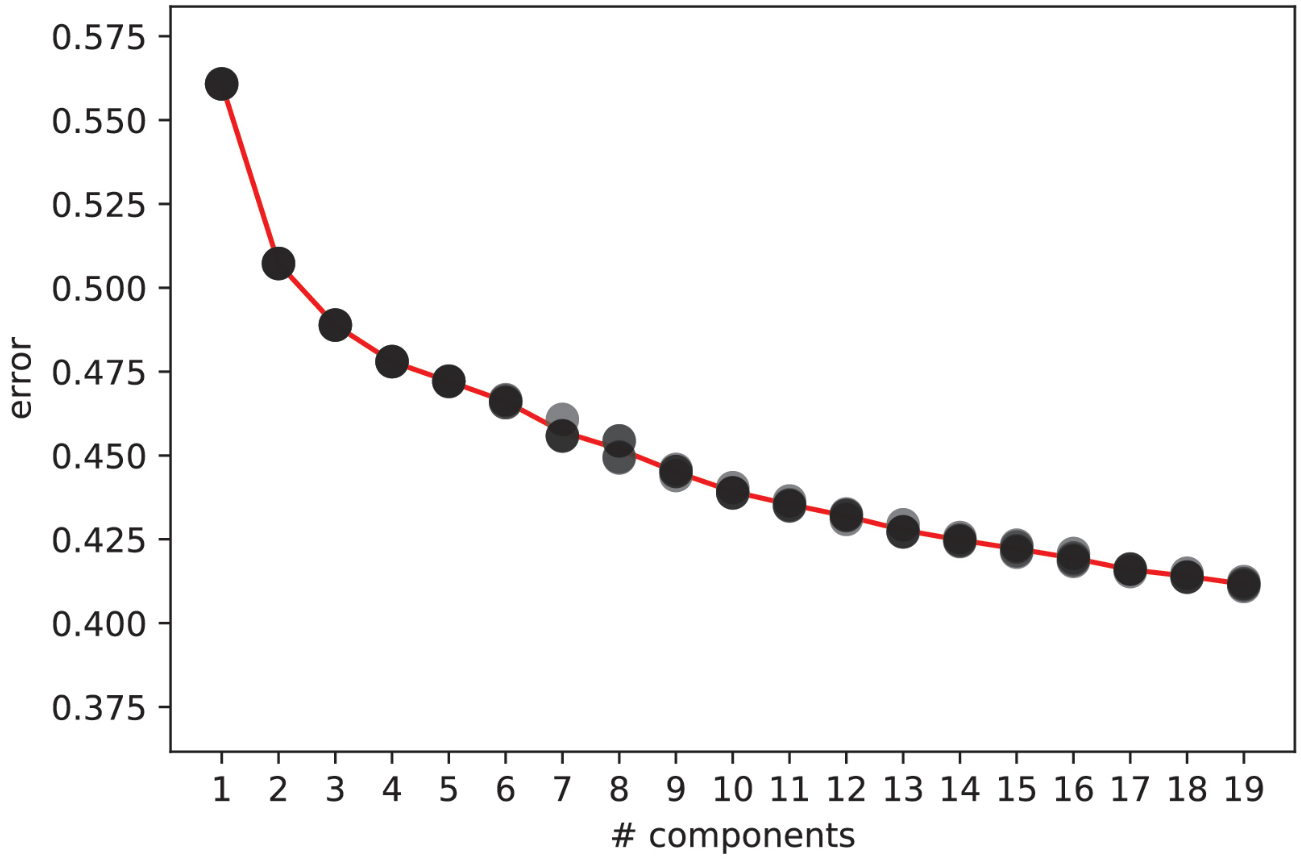

Figure S1. Reconstruction errors for TCA. Corresponding reconstruction error of demixed source models for varying R. For each source count from 1 to 19, four independent demixings are trained to the data. Four models are trained for each value of R (gray circles) and plotted with their average errors (red curve).

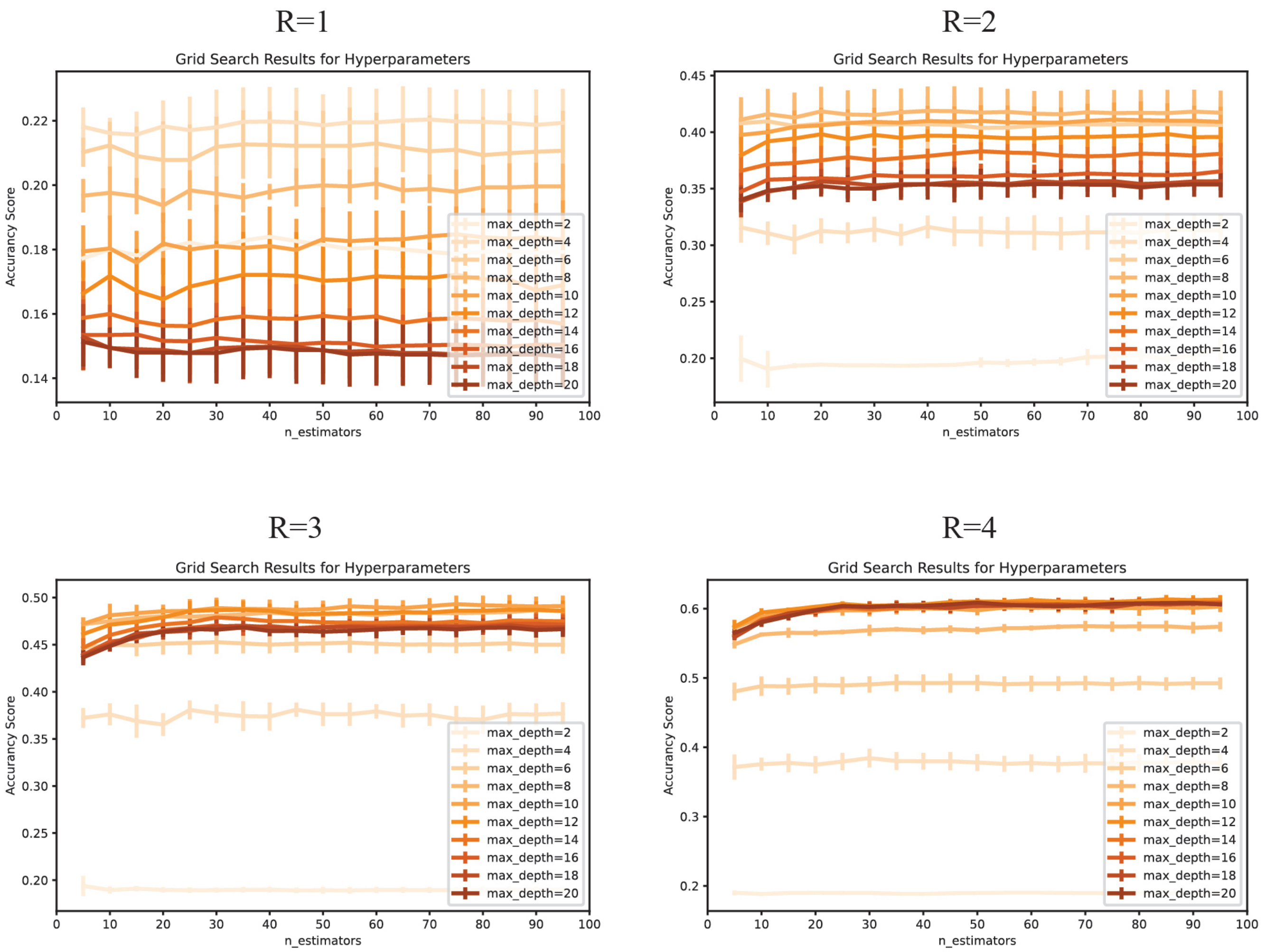

Figure S2. Results of random forest grid search. Grid search results for random forest classifiers are shown for multiple hyperparameter values of *n_estimators* and *max_depth*. The highest performing hyperparameter pairs are chosen (for R=1,2,3, and 4) from this grid search.

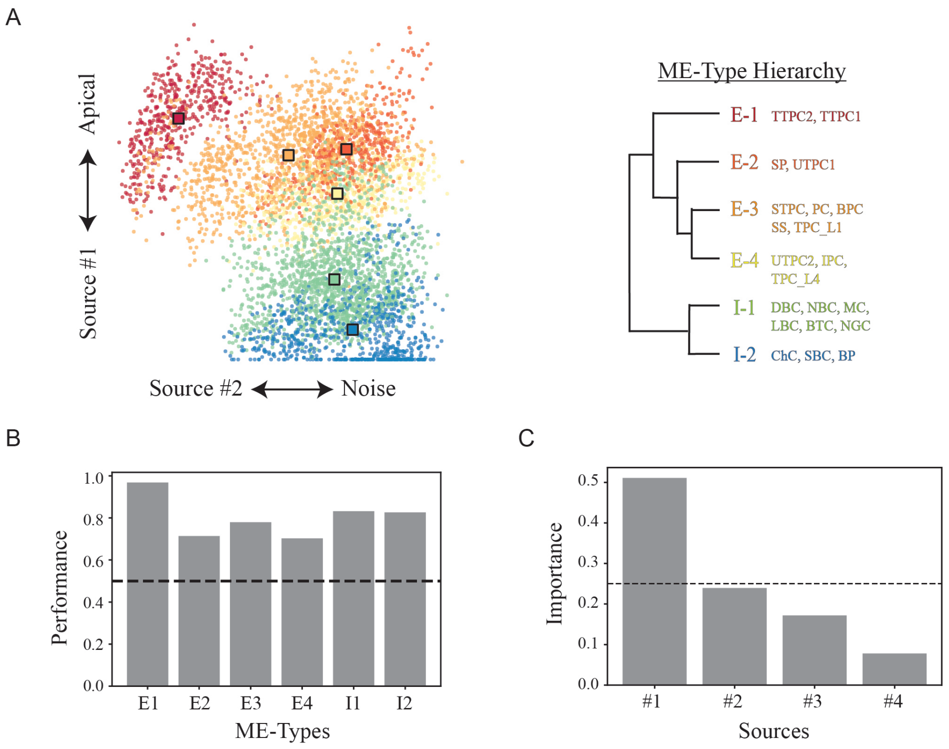

Figure S3. Relationships between model morpho-electrophysiological types and demixed EAP sources. Each point in the scatter plot shows the SU source prevalences for two dominant EAP sources across all single units grouped by ME-types (colored circles) similar to Figure 7. The expected demixed representation of each ME-type (colored squares) are depicted by the bootstrapped centroids using 1000 random subsamples. The ME-type hierarchy is depicted on the right and identical to Figure 7. (B) Average prediction accuracy for random forest classifier (out-of-bag score) shown for each ME-type. Dashed line depicts 50% threshold. (C) Feature importances estimated by random forest classifier using 100 random subsamples of simulated units. Dashed line depicts the threshold for equally weighted importances. 95% confidence intervals were too small to show.

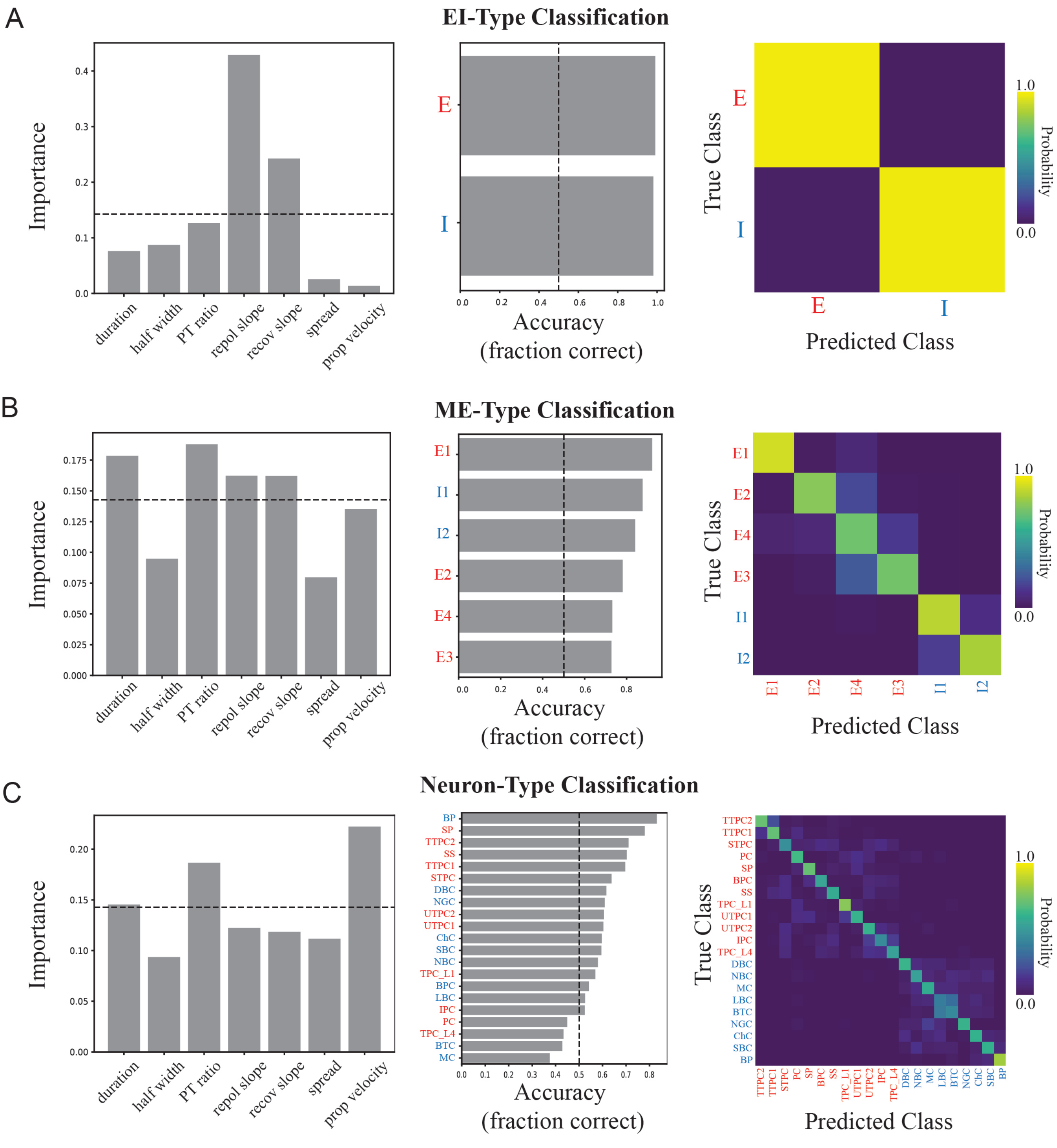

Figure S4. Classification using established feature-based approach. To assess the performance of the four source model compared to a feature-based approach, random forest classifiers were trained for each of the classifications (EI-type, ME-type, and neuron-type) using five single channel features and two multi-channel features. (A) EI-type classification results. (Left) Feature importances estimated by random forest classifier. Dashed line depicts the threshold for equally weighted importances. (Middle) Average prediction accuracy for random forest classifier (out-of-bag score) for E- and I-types. Dashed line depicts 50% threshold. (Right) Confusion matrix from random forest classifier across all types. Class labels show excitatory (red) and inhibitory (blue) types. (B) Same as top row but for ME-type classification. (C) Same as top row but for neuron-type classification.

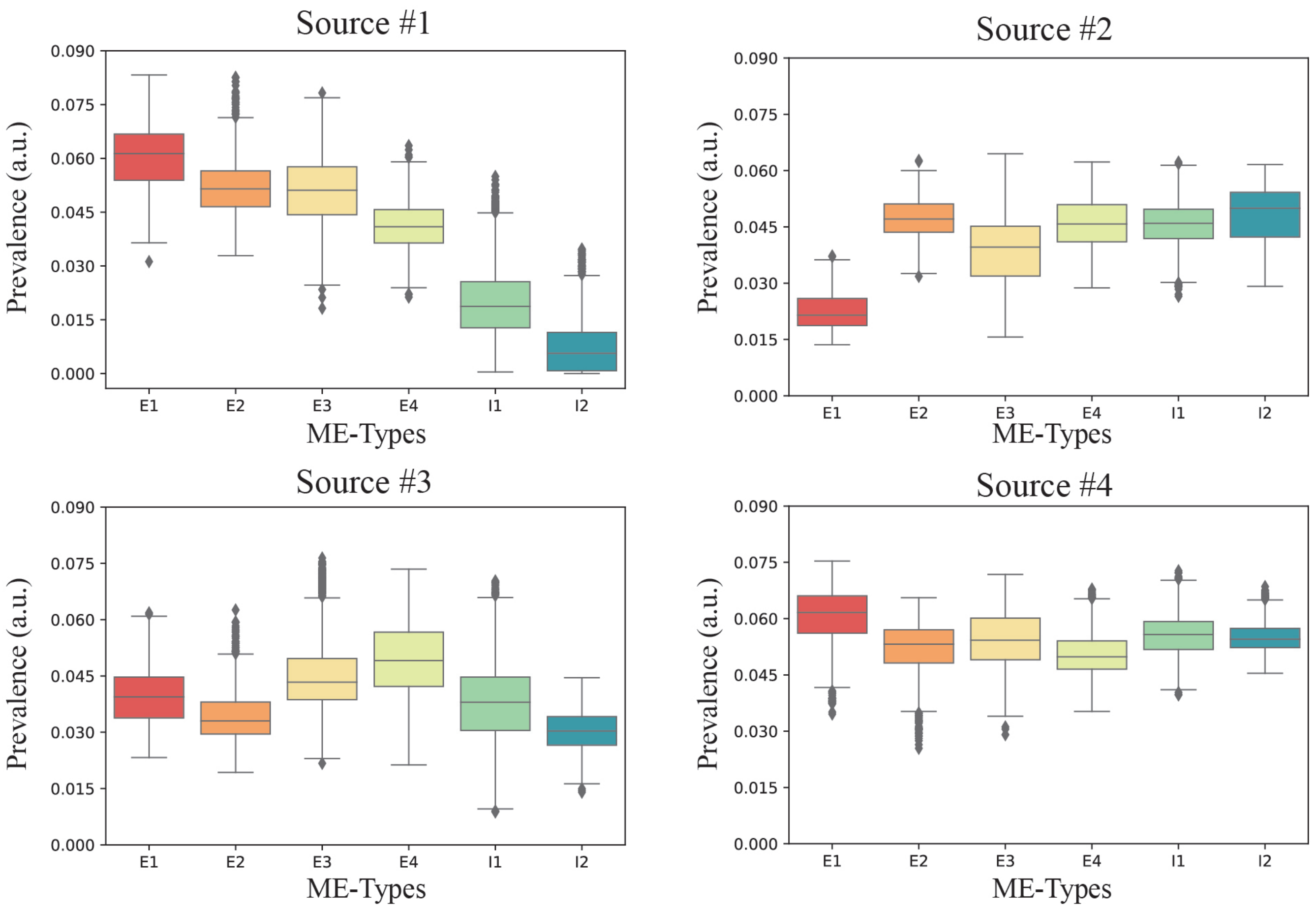

Figure S5. Source prevalences for each ME-type. Box-and-whisker plots show the relative prevalence of the different sources for the ME-types. The middle line of each box depicts the median of the distribution for that ME-type, each box corresponds to upper and lower quartiles, and whisker lines show maximum/minimum values of distribution once outliers are found (grey diamonds).

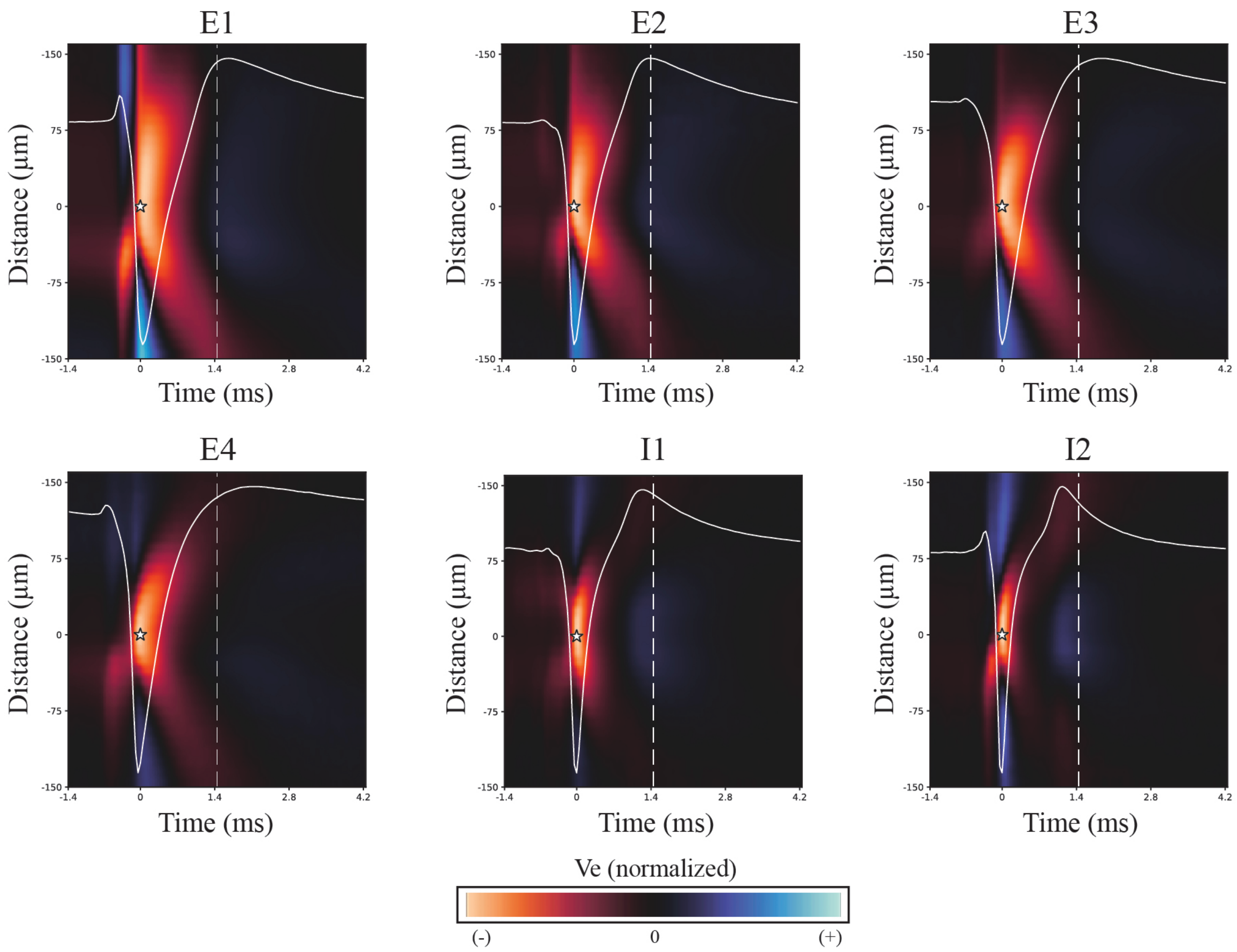

Figure S6. Example spatio-temporal EAPs for each ME-type. Colormaps show average EAP waveforms across all units corresponding to each ME-type. EAPs were normalized before averaging. Color scale adjusted to the maximum fluctuation of the EAP. Star indicates the center channel and the intracellular spike time for all units. Center waveform depicted in white.
